# Supplementary material for: Foraging, Fear and Behavioral Variation in a Traplining Hummingbird
Source: Animals (Basel). 2023 Jun 15;13(12):1997. doi: 10.3390/ani13121997 (PMC10295738; doi:10.3390/ani13121997)
Supplement: Supplementary file 1 [file animals-13-01997-s001.zip › supplementary_S1.html]

 
  

 
 

 

 
 

 LBH foraging efficiency 
 


 
 
 
 
 
 
 
 
 
 

 
 

   

 
 

 

 
 
   
     Table of contents 
   
   
     1   Load packages  
     2   Load data and set parameters  
     3   Variable description ( only those in bold were used in the statistical analysis ):  
     4   Exploring data  
     5   Repeatability  
     6   MCMCglmm mixed-effect models 
   
     6.1   Single predictor models  
     6.2   Model selection results  
     6.3   Single global model  
     6.4   Diagnostic stats and plots on MCMCglmm models  
    
   
 
 
 
 
 

 
 
   LBH foraging efficiency     Code    Show All Code    Hide All Code       View Source     
 Statistical analysis 
 


 

     
     Author 
     
               Marcelo Araya-Salas, PhD   
           
   
    
     
     Published 
     
       June 4, 2023 
     
   
  
    
   
  

 

 
 
 
 

 
 
 
 Statistical analysis for the paper: 
 
 Wojczulanis-Jakubas, K.; Araya-Salas, M. Foraging, Fear and Behavioral Variation in a Traplining Hummingbird. Animals 2023, 13, x. https://doi.org/10.3390/xxxxx 
 
 
   
 
  1  Load packages 
 
 
 Code 
       ## add 'developer/' to packages to be  
    ## installed from github  
   x  <-   c ( "viridis" ,  "readxl" ,  "ggplot2" ,  "tidyverse" , 
        "lmerTest" ,  "lme4" ,  "smatr" ,  "ggpubr" ,  "MCMCglmm" , 
        "corrplot" ,  "rptR" ,  "pbapply" ,  "MuMIn" ,  "parallel" , 
        "kableExtra" ,  "ggridges" ,  "cowplot" ,  "ggplotify" , 
        "gridExtra" ,  "grid" ) 
    
   sketchy ::  load_packages (x)        
 
 
 
 
  2  Load data and set parameters 
 
 
 Code 
      cols  <-   viridis ( 10 ,  alpha =   0.6 ) 
    
    # function to get posterior estimates within  
    # the HPD interval  
   HPD_mcmc  <-   function (y,  long =   TRUE ) { 
    
       out  <-   lapply ( 1  :  ncol (y),  function (x) { 
            # calculate hpd  
           hpd  <-   HPDinterval (y[, x]) 
    
            # get sol as vector  
           vctr  <-  y[, x] 
    
            # clip vector to hpd range  
           hpdmcmc  <-  vctr[vctr  >  hpd[ 1 ]  &  vctr  <  
               hpd[ 2 ]] 
    
            return (hpdmcmc) 
       }) 
    
        # get them together  
       hpd.mcmcs  <-   do.call (cbind, out) 
    
        # change colnames  
        colnames (hpd.mcmcs)  <-   colnames (y) 
    
        # put it in long format  
        if  (long) { 
           est.df  <-   lapply ( 1  :  ncol (hpd.mcmcs),  function (x) { 
    
                data.frame ( predictor =   colnames (hpd.mcmcs)[x], 
                    effect_size =  hpd.mcmcs[, x], 
                    stringsAsFactors =   FALSE ) 
           }) 
    
            # get them together  
           hpd.mcmcs  <-   do.call (rbind, est.df) 
       } 
    
        return (hpd.mcmcs) 
   } 
    
    # color for corrplot  
   col.crrplt  <-   colorRampPalette ( c (cols[ 1  :  2 ],  rep ( "white" , 
        1 ), cols[ 6  :  7 ]))( 100 ) 
    
    
    # plot diagonostic stuff for mcmcglmmm  
    # models  
   plot_repl_mcmc_models  <-   function (X,  pal =  viridis, 
        begin =   0.1 ,  end =   1 ) { 
    
        # extract mcmc chains  
       sol_l  <-   lapply (X,  "[[" ,  "Sol" ) 
    
        # put them in a single matrix  
       sol_mat  <-   do.call (cbind, sol_l) 
    
        colnames (sol_mat)  <-   paste0 ( colnames (sol_mat), 
            " repl" ,  rep ( 1  :  length (X),  each =   ncol (sol_l[[ 1 ]]))) 
    
       sol_mat  <-  sol_mat[,  order ( colnames (sol_mat))] 
    
        # add class attributes of MCMC chains  
        class (sol_mat)  <-   "mcmc"  
        attr (sol_mat,  "mcpar" )  <-   attr (X[[ 1 ]] $ Sol, 
            "mcpar" ) 
    
        # extract each column as a mcmc matrix  
       mcmcs  <-   lapply ( seq_len ( ncol (sol_mat)),  function (x) sol_mat[, 
           x,  drop =   FALSE ]) 
    
       cols  <-   pal ( length (mcmcs),  alpha =   0.7 ,  begin =  begin, 
            end =  end) 
    
        # test colors plot(1:length(cols), col =  
        # cols, pch = 20, cex =4)  
    
        for  (y  in   1  :  length (mcmcs)) { 
    
            # trace and density  
            plot (mcmcs[[y]],  col =  cols[y]) 
    
       } 
    
        # autocorrelation  
        par ( mfrow =   c ( 1 ,  2 )) 
        for  (y  in   1  :  length (mcmcs)) { 
    
            autocorr.plot (mcmcs[[y]],  col =  cols[y], 
                lwd =   4 ,  ask =   FALSE ,  auto.layout =   FALSE ) 
       } 
    
        par ( mfrow =   c ( 1 ,  1 )) 
        ## add global plots and gelman test  
        ## gelman_diagnostic  
       gel_diag  <-   as.data.frame ( gelman.diag ( mcmc.list (sol_l)) $ psrf) 
    
        # add estimate as column  
       gel_diag $ estimate  <-   rownames (gel_diag) 
    
        # reorder columns  
       gel_diag  <-  gel_diag[,  c ( 3 ,  1 ,  2 )] 
    
        # plot table  
        grid.newpage () 
        grid.draw ( tableGrob (gel_diag,  rows =   NULL , 
            theme =   ttheme_default ( base_size =   25 ))) 
   } 
    
    
    ##### chunk output stuff  
   knitr :: opts_chunk $  set ( dpi =   58 ,  fig.width =   12 , 
        fig.height =   8 ) 
    
    # ggplot2 theme  
    theme_set ( theme_classic ( base_size =   30 ,  base_family =   "Arial" )) 
    
    
    ## data  
   foraging_data  <-  ff  <-   read_excel ( "./data/raw/ff.xlsx" ) 
    
    names (foraging_data)[ names (foraging_data)  ==   "ID.." ]  <-   "indiv"         
 
 
 

 
 
 
  3  Variable description ( only those in bold were used in the statistical analysis ): 
 
 abs_nflo: absolute number of feeders used (e.g. feeder: A, B, A, B; abs_nflo = 2) 
 nflo_chang: number of feeders changes (e.g. feeder: A, B, A, B; nflo_chang = 3) 
 nouts: number of “OUTs” foraging breaks (i.e. bill NOT inserted in the feeder) 
 nins: number of “INs” - foraging intervals (i.e. bill inserted in the feeder) 
 mean_durins: mean duration of “INs” 
 
 tot_durins: total duration of the “INs” (i.e. sum of all ins) 
 mean_durouts: mean duration of “OUTs” 
 
 tot_durouts: total duration of the “OUTs” (i.e. sum of all ins) 
 
 tot_durfor: total duration of foraging visit (i.e .time between the very first insert and the end of the visit) mov_totdist: total distance covered during the foraging visit 
 mov_spead: total distance covered during the foraging visit divided by the total duration of the visit 
 mov_feroc: coeficient of variance for the birds position in the 2D space 
 ID..: birds ID 
 stan_nflochang: time-standardized nflo_change 
 stan_nouts: time-standardized nouts 
 stan_nins: time-standardized nins 
 stand_totdist: time-standardized totdist 
  stan_nflo: time-standardized abs_nflo (i.e. abs_nflo/tot_durfor)  (PROXY FOR EXPLORATIONS)    
 
  for_eff: foraging efficency, i.e. tot_durins / totdurfor  
  Latency: latency to approach the feeder (i.e. time between birds appearance, like the first hovering in front of the feeder and onset of the visit)  (PROXY FOR RISK AVOIDANCE)    
 
  mov_feroc_stand: time-standardized mov_feroc  (PROXY FOR AROUSAL)    
 
 
 
  4  Exploring data 
 
 
 Code 
       # target variables  
   vars  <-   c ( "stan_nflo" ,  "for_eff" ,  "Latency" ,  "mov_feroc_stand" ) 
    
    # look at data distribution  
   long_foragin_data  <-   do.call (rbind,  lapply (vars, 
        function (x)  data.frame ( var =  x,  value =  foraging_data[, 
            names (foraging_data)  ==  x,  drop =   TRUE ]))) 
    
    ggplot (long_foragin_data,  aes (var, value))  +   geom_violin ( fill =  cols[ 9 ])  +  
        coord_flip ()  +   ggtitle ( "Raw parameters" )  +  
        labs ( x =   "Parameter" ,  y =   "Raw value" )        
 
 
   
 
 
 Code 
       # log transformed  
    ggplot (long_foragin_data,  aes (var,  log (value  +  
        1 )))  +   geom_violin ( fill =  cols[ 9 ])  +   coord_flip ()  +  
        ggtitle ( "Log-transformed parameters" )  +   labs ( x =   "Parameter" , 
        y =   "Log value" )        
 
 
   
 
 
 Code 
       # log transform variables  
   foraging_data $ arousal  <-   log (foraging_data $ mov_feroc_stand  +  
        1 ) 
   foraging_data $ exploration  <-   log (foraging_data $ stan_nflo  +  
        1 ) 
   foraging_data $ risk_avoidance  <-   log (foraging_data $ Latency  +  
        1 ) 
   foraging_data $ foraging_efficiency  <-   log (foraging_data $ for_eff  +  
        1 ) 
    
   foraging_data $ context  <-   ifelse (foraging_data $ treat  ==  
        "Ctr" ,  "Low risk" ,  "High risk" ) 
    
   foraging_data $ context  <-   factor (foraging_data $ context, 
        levels =   c ( "Low risk" ,  "High risk" )) 
    
    # new target variables  
   vars  <-   c ( "exploration" ,  "risk_avoidance" ,  "arousal" ) 
    
    # correlation matrix  
   cm  <-   cor (foraging_data[, vars],  use =   "pairwise.complete.obs" ) 
    
    # visualize collinearity  
    corrplot.mixed (cm,  upper =   "ellipse" ,  lower =   "number" , 
        tl.pos =   "lt" ,  upper.col =  col.crrplt,  lower.col =  col.crrplt, 
        tl.col =   "black" ,  tl.cex =   2 )        
 
 
   
 
 
 
 
  Long right tails in distributions (better to log!)  
  Personality parameters were log-tranformed and renamed: 
 
 log(stan_nflo) ->  exploration  
 
 log(for_eff) ->  foraging_efficiency  
 Log(Latency) ->  risk_avoidance  
 Log(mov_feroc_stand) ->  arousal  
  
  Little collinearity between predictors  
 
 
 
 
  5  Repeatability 
 
 
 Code 
       pboptions ( type =   "none" ) 
    # rep movement  
    
   rpt_arousal  <-   rpt (arousal  ~  ( 1   |  indiv),  data =  foraging_data[foraging_data $ context  ==  
        "Low risk" , ],  grname =   "indiv" ,  nboot =   100 , 
        npermut =   100 ,  parallel =   TRUE ) 
    
   rpt_exploration  <-   rpt (exploration  ~  ( 1   |  indiv), 
        data =  foraging_data[foraging_data $ context  ==  
            "Low risk" , ],  grname =   "indiv" ,  nboot =   100 , 
        npermut =   100 ,  parallel =   TRUE ) 
    
   rpt_risk  <-   rpt (risk_avoidance  ~  ( 1   |  indiv), 
        data =  foraging_data[foraging_data $ context  ==  
            "Low risk" , ],  grname =   "indiv" ,  nboot =   100 , 
        npermut =   100 ,  parallel =   TRUE ) 
    
   rpt_foraging_efficiency  <-   rpt (foraging_efficiency  ~  
       ( 1   |  indiv),  data =  foraging_data[foraging_data $ context  ==  
        "Low risk" , ],  grname =   "indiv" ,  nboot =   100 , 
        npermut =   100 ,  parallel =   TRUE ) 
    
    
    saveRDS ( list ( arousal =  rpt_arousal,  exploration =  rpt_exploration, 
        risk_avoidance =  rpt_risk,  foraging_efficiency =  rpt_foraging_efficiency), 
        "./output/Repeatability results.RDS" )        
 
 
 
 
 Code 
      rept  <-   readRDS ( "./output/Repeatability results.RDS" ) 
    
   reps  <-   lapply ( 1  :  length (rept),  function (x) { 
    
       X  <-  rept[[x]] 
        data.frame ( param =   names (rept)[x],  R =  X $ R[ 1 , 
           ],  low.CI =  X $ CI_emp[ 1 ,  1 ],  hi.CI =  X $ CI_emp[ 1 , 
            2 ]) 
   }) 
    
   reps.df  <-   do.call (rbind, reps) 
    
    ggplot (reps.df,  aes ( x =  param,  y =  R))  +   geom_hline ( yintercept =   0 , 
        lty =   2 )  +   geom_point ( col =  cols[ 7 ],  size =   5 )  +  
        geom_errorbar ( aes ( ymin =  low.CI,  ymax =  hi.CI), 
            width =   0 ,  col =  cols[ 7 ],  size =   2 )  +  
        coord_flip ()  +   labs ( y =   "Repeatability" ,  x =   "Parameters" )        
 
 
   
 
 
 
 
  Medium to low repeatability  
  Non-significant repeatability for arousal  
 
 
 
 
  6  MCMCglmm mixed-effect models 
 Bayesian MCMC generalized linear models to predict foraging efficiency with personaltiy-related parameters and their interaction with context (low or high risk) as predictors and individual as a random effect. 
 We used two modeling approaches. In the first one ( “single predictor approach” ) indenpendent model selection procedures were run for each personality-parameter. In the second approach a  single global model  containing all 3 interactions was compared against submodels containing 1 and 2 interaction. In both cases all model selection procedures included the “classical” hypothesis model that ignores within individual variation (so only risk level as predictor). 
  -->
 
 
  6.1  Single predictor models 
 Three models were compared for each parameter: 
 
 only context as predictor (i.e.  “classical” hypothesis ): 
 
  \[foraging\ efficiency \sim context + (1 | indiv)\]  
 
 context, personality parameters and their interaction as predictors ( alternative hypothesis accounting for individual differences ): 
 
  \[foraging\ efficiency \sim context * personality\ parameter + (1 | indiv)\]  
 
 Null model with no predictor: 
 
  \[foraging\ efficiency \sim 1 + (1 | indiv)\]  
 
  -->
 A loop is used to run these 3 models for each selected acoustic parameters. Each model is replicated 3 times with starting values sampled from a Z-distribution (“start” argument in MCMCglmm()) and mean-centered so intercept is found at the mean of the predictor variable. Parameters are scaled (i.e. z-transformed) to obtained standardized effect sizes (within the loop). Diagnostic plots for MCMC model performance are shown at the end of this report: 
 
 
 Code 
      itrns  <-   1e+05  
   burnin  <-   10000  
    # null model  
   mcmc_output  <-   pblapply ( c ( "arousal" ,  "exploration" , 
        "risk_avoidance" ),  cl =   detectCores ()  -   1 , 
        function (x) { 
    
           foraging_subdata  <-  foraging_data[,  c (x, 
                "indiv" ,  "foraging_efficiency" ,  "context" )] 
    
           foraging_subdata  <-  foraging_subdata[ complete.cases (foraging_subdata[, 
               x]), ] 
    
            # mean centering  
           foraging_subdata[, x]  <-  foraging_subdata[, 
               x]  -   mean (foraging_subdata[, x,  drop =   TRUE ], 
                na.rm =   TRUE ) 
    
           md_null  <-   replicate ( 3 ,  MCMCglmm ( formula ( "foraging_efficiency ~ 1" ), 
                random =   ~ indiv,  data =  foraging_subdata, 
                verbose =   FALSE ,  nitt =  itrns,  start =   list ( QUASI =   FALSE ), 
                burnin =  burnin),  simplify =   FALSE ) 
    
           md_only_context  <-   replicate ( 3 ,  MCMCglmm ( formula ( "foraging_efficiency ~ context" ), 
                random =   ~ indiv,  data =  foraging_subdata, 
                verbose =   FALSE ,  nitt =  itrns,  start =   list ( QUASI =   FALSE ), 
                burnin =  burnin),  simplify =   FALSE ) 
    
           md_only_parameter  <-   replicate ( 3 ,  MCMCglmm ( formula ( paste ( "foraging_efficiency ~" , 
               x)),  random =   ~ indiv,  data =  foraging_subdata, 
                verbose =   FALSE ,  nitt =  itrns,  start =   list ( QUASI =   FALSE ), 
                burnin =  burnin),  simplify =   FALSE ) 
    
           md_interation  <-   replicate ( 3 ,  MCMCglmm ( formula ( paste ( "foraging_efficiency ~ context *" , 
               x)),  random =   ~ indiv,  data =  foraging_subdata, 
                verbose =   FALSE ,  nitt =  itrns,  start =   list ( QUASI =   FALSE ), 
                burnin =  burnin),  simplify =   FALSE ) 
    
            # put together the first models  
           msDIC  <-   model.sel (md_null[[ 1 ]], md_only_context[[ 1 ]], 
               md_only_parameter[[ 1 ]], md_interation[[ 1 ]], 
                rank =   "DIC" ) 
    
            # rename delta and weight  
            names (msDIC)[ names (msDIC)  %in%   c ( "delta" , 
                "weight" )]  <-   paste0 ( "DIC." ,  c ( "delta" , 
                "weight" )) 
    
            # put together the first models  
           msAIC  <-   model.sel (md_null[[ 1 ]], md_only_context[[ 1 ]], 
               md_only_parameter[[ 1 ]], md_interation[[ 1 ]], 
                rank =   "AIC" ) 
    
            # rename delta and weight  
            names (msAIC)[ names (msAIC)  %in%   c ( "delta" , 
                "weight" )]  <-   paste0 ( "AIC." ,  c ( "delta" , 
                "weight" )) 
    
           ms  <-   cbind (msDIC, msAIC[,  c ( "AIC" ,  "AIC.delta" , 
                "AIC.weight" )]) 
    
            # rename rows so they match  
            # predictor names  
            rownames (ms)  <-   gsub ( "[[1]]" ,  "" ,  rownames (ms), 
                fixed =   TRUE ) 
    
            # save models in a list  
           res  <-   list ( model.tab =  ms,  md_only_context =  md_only_context, 
                md_only_parameter =  md_only_parameter, 
                md_interation =  md_interation,  md_null =  md_null) 
       }) 
    
    names (mcmc_output)  <-   c ( "arousal" ,  "exploration" , 
        "risk_avoidance" ) 
    
    saveRDS (mcmc_output,  "model_selection_predict_foraging_efficiency.RDS" )        
 
 
 
 
  6.2  Model selection results 
 
 ordered by delta DIC (but AIC produces equivalent results) 
 best model for each parameters is highlighted in green 
 
 
 
 Code 
      mcmc_output  <-   readRDS ( "./output/model_selection_predict_foraging_efficiency.RDS" ) 
    
    # put all model selection results in a list  
   mod.list  <-   lapply ( 1  :  length (mcmc_output),  function (i)  data.frame ( response =   names (mcmc_output)[i], 
        predictors =   rownames (mcmc_output[[i]][[ 1 ]]), 
        as.data.frame (mcmc_output[[i]][[ 1 ]])[,  5  :  12 ], 
        stringsAsFactors =   FALSE )) 
    
    # make a data frame with all results  
   mod.sel.tab  <-   do.call (rbind, mod.list) 
    
    # rename predictors for table  
   mod.sel.tab $ predictors[ grep ( "interation" , mod.sel.tab $ predictors)]  <-   "Context interaction"  
   mod.sel.tab $ predictors[ grep ( "null" , mod.sel.tab $ predictors)]  <-   "Null"  
   mod.sel.tab $ predictors[ grep ( "only_context" , mod.sel.tab $ predictors)]  <-   "Context"  
   mod.sel.tab $ predictors[ grep ( "only_parameter" , 
       mod.sel.tab $ predictors)]  <-   "Parameter"  
    
    
   mod.sel.tab $ DIC.delta  <-   round (mod.sel.tab $ DIC.delta, 
        2 ) 
   mod.sel.tab $ DIC.weight  <-   round (mod.sel.tab $ DIC.weight, 
        2 ) 
   mod.sel.tab $ AIC.delta  <-   round (mod.sel.tab $ AIC.delta, 
        2 ) 
   mod.sel.tab $ AIC.weight  <-   round (mod.sel.tab $ AIC.weight, 
        2 ) 
    
    options ( knitr.kable.NA =   "" ) 
    
   df1  <-  knitr ::  kable (mod.sel.tab[,  c ( "response" , 
        "predictors" ,  "df" ,  "DIC" ,  "DIC.delta" ,  "DIC.weight" , 
        "AIC" ,  "AIC.delta" ,  "AIC.weight" )],  row.names =   FALSE , 
        escape =   FALSE ,  format =   "html" ) 
    
   df1  <-   row_spec (df1,  which (mod.sel.tab $ DIC.delta  ==  
        0 ),  background =   adjustcolor (cols[ 9 ],  alpha.f =   0.3 )) 
    
    kable_styling (df1,  bootstrap_options =   c ( "striped" , 
        "hover" ,  "condensed" ,  "responsive" ),  full_width =   FALSE , 
        font_size =   15 )        
 
 

 
  
   
     response  
     predictors  
     df  
     DIC  
     DIC.delta  
     DIC.weight  
     AIC  
     AIC.delta  
     AIC.weight  
   
  
 
   
     arousal  
     Context interaction  
     6  
     -365.8347  
     0.00  
     1.00  
     -365.8307  
     0.00  
     1.00  
   
   
     arousal  
     Parameter  
     4  
     -328.9776  
     36.86  
     0.00  
     -331.0490  
     34.78  
     0.00  
   
   
     arousal  
     Context  
     4  
     -309.6083  
     56.23  
     0.00  
     -312.2369  
     53.59  
     0.00  
   
   
     arousal  
     Null  
     3  
     -298.5859  
     67.25  
     0.00  
     -302.1584  
     63.67  
     0.00  
   
   
     exploration  
     Context interaction  
     6  
     -348.0369  
     0.00  
     1.00  
     -348.9852  
     0.00  
     1.00  
   
   
     exploration  
     Context  
     4  
     -310.8631  
     37.17  
     0.00  
     -313.1746  
     35.81  
     0.00  
   
   
     exploration  
     Parameter  
     4  
     -307.5661  
     40.47  
     0.00  
     -310.6167  
     38.37  
     0.00  
   
   
     exploration  
     Null  
     3  
     -298.6007  
     49.44  
     0.00  
     -302.1654  
     46.82  
     0.00  
   
   
     risk_avoidance  
     Parameter  
     4  
     -314.1987  
     0.00  
     0.53  
     -316.4061  
     0.00  
     0.72  
   
   
     risk_avoidance  
     Context interaction  
     6  
     -313.7740  
     0.42  
     0.43  
     -314.0783  
     2.33  
     0.23  
   
   
     risk_avoidance  
     Context  
     4  
     -308.9691  
     5.23  
     0.04  
     -311.0324  
     5.37  
     0.05  
   
   
     risk_avoidance  
     Null  
     3  
     -296.4492  
     17.75  
     0.00  
     -299.8973  
     16.51  
     0.00  
   
 
 

 
 
 
 
  All best models contained an interaction with a personality parameter  
  All models with interaction provided a better fit than the context (low vs high risk) models  
 
 
 Plot effect sizes by response variable (only models that improved fit compared to the null models are evaluated): 
 
 
 Code 
       # select best models based on BIC  
   best_mods  <-   lapply (mcmc_output,  function (X){  
      
      # if best model was at least 2 BIC units higher than null  
      if  (X[[ 1 ]][ "md_null" ,  "DIC.delta" ]  >   2 )  
        return (X[[  rownames (X[[ 1 ]])[ 1 ] ]][[ 1 ]])  else  
          return ( NA )  # else if models were as good as null model return NA  
     }) 
    
    # rename  
    names (best_mods)  <-   names (mcmc_output) 
    
    # remove the NA ones (the ones in which the null model was the best)  
   best_mods  <-  best_mods[ sapply (best_mods, class)  ==   "MCMCglmm" ] 
      
    # extract fixed effect size  
   out  <-   lapply ( 1  :  length (best_mods),  function (x){ 
      
      # fixed effects  
     fe  <-   summary (best_mods[[x]]) $ solutions 
    
      # Confidence intervals  
     ci  <-   HPDinterval (best_mods[[x]] $ Sol) 
      
      # sample sizes    
     obs  <-  foraging_data[ complete.cases (foraging_data[ ,  names (best_mods)[x]]), ] 
      
      # put results together in a data frame  
     res  <-   data.frame ( 
        stringsAsFactors =   FALSE ,  
        # response variable name  
        response =   "foraging effiency" ,  
        # personality parameter  
        parameter =   names (best_mods)[x], 
        # predictor name  
        predictor =   rownames (ci)[ 2  :  nrow (ci)],  
        effect_size =  fe[ -  1 ,  "post.mean" ],  
        # lower confident interval  
        CI_2.5 =  ci[ 2  :  nrow (ci),  1 ],  
        # upper confident interval  
        CI_97.5 =  ci[ 2  :  nrow (ci),  2 ],  
        # p value  
        pMCMC  =  fe[ -  1 ,  "pMCMC" ],  
        #intercept  
        intercept =  fe[ 1 ,  "post.mean" ], 
        # number of individuals  
        n.indv =   length ( unique (obs $ indiv)),  
        # number of observations  
        n.obs =   nrow (obs),  
        # mean response  
        mean =   mean (obs[,  names (best_mods)[x],  drop =   TRUE ],  na.rm =   TRUE ),  
        # standard deviation of response  
        sd =   sd (obs[,  names (best_mods)[x],  drop =   TRUE ],  na.rm =   TRUE ) 
       ) 
      
     return (res) 
   }) 
    
    # put effect sizes in a single data frame   
   effect_size_single_preds  <-   do.call (rbind, out) 
    rownames (effect_size_single_preds)  <-   1  :  nrow (effect_size_single_preds) 
    
    
   md  <-  effect_size_single_preds[,  !  grepl ( "mean|sd" ,  names (effect_size_single_preds))] 
    
   md $ CI_2 .5   <-   round (md $ CI_2 .5 ,  4 ) 
   md $ CI_97 .5   <-   round (md $ CI_97 .5 ,  4 ) 
    
    # get the ones that do not overlap with 0  
   mltp  <-  md $ CI_2 .5   *  md $ CI_97 .5  
    
   md $ CI_2 .5   <-   ifelse (mltp  >   0 ,  cell_spec (md $ CI_2 .5 ,  "html" ,  color =  "white" ,  background =  cols[ 7 ],  bold =  T,   font_size =   12 ),   cell_spec (md $ CI_2 .5 ,  "html" )) 
    
   md $ CI_97 .5   <-   ifelse (mltp  >   0 ,  cell_spec (md $ CI_97 .5 ,  "html" ,  color =  "white" ,  background =  cols[ 7 ],  bold =  T,   font_size =   12 ),   cell_spec (md $ CI_97 .5 ,  "html" )) 
    
   df1  <-  knitr ::  kable (md,  row.names =   FALSE ,  escape =   FALSE ,  format =   "html" ,  digits =   c ( 4 )) 
    
   df1  <-   row_spec (df1,  which (mltp  >   0 ),  background =   adjustcolor (cols[ 9 ],  alpha.f =   0.3 )) 
      
    kable_styling (df1,  bootstrap_options =   c ( "striped" ,  "hover" ,  "condensed" ,  "responsive" ),  full_width =   FALSE ,  font_size =   12 )        
 
 

 
  
   
     response  
     parameter  
     predictor  
     effect_size  
     CI_2.5  
     CI_97.5  
     pMCMC  
     intercept  
     n.indv  
     n.obs  
   
  
 
   
     foraging effiency  
     arousal  
     contextHigh risk  
     -0.0352  
      -0.0668   
      -0.0021   
     0.0347  
     0.5343  
     12  
     193  
   
   
     foraging effiency  
     arousal  
     arousal  
     0.0663  
      0.0195   
      0.1072   
     0.0044  
     0.5343  
     12  
     193  
   
   
     foraging effiency  
     arousal  
     contextHigh risk:arousal  
     0.2815  
      0.1853   
      0.3802   
     0.0001  
     0.5343  
     12  
     193  
   
   
     foraging effiency  
     exploration  
     contextHigh risk  
     -0.0645  
      -0.0972   
      -0.0316   
     0.0002  
     0.5346  
     12  
     193  
   
   
     foraging effiency  
     exploration  
     exploration  
     0.3039  
      0.034   
      0.5697   
     0.0296  
     0.5346  
     12  
     193  
   
   
     foraging effiency  
     exploration  
     contextHigh risk:exploration  
     -1.1133  
      -1.4827   
      -0.7451   
     0.0001  
     0.5346  
     12  
     193  
   
   
     foraging effiency  
     risk_avoidance  
     risk_avoidance  
     -0.0648  
      -0.0924   
      -0.0377   
     0.0001  
     0.5209  
     11  
     192  
   
 
 

 
 
 
  6.2.1  Effect sizes (on foraging efficiency) for interaction terms 
 
 
 Code 
       # get high prob density intervals  
   hpd.mcmc.l  <-   lapply ( 1  :  length (best_mods),  function (x) { 
    
       hpd.mcmcs  <-   HPD_mcmc (best_mods[[x]] $ Sol) 
    
        return (hpd.mcmcs) 
   }) 
    
   hpd.mcmcs  <-   do.call (rbind, hpd.mcmc.l) 
    
    # remove ohter parameters  
   hpd.mcmcs  <-  hpd.mcmcs[ grep ( "risk$" , hpd.mcmcs $ predictor, 
        invert =   TRUE ), ] 
    
    # context model  
   contextHDP  <-   HPD_mcmc (mcmc_output $ arousal $ md_only_context[[ 1 ]] $ Sol) 
    
   hpd.mcmcs  <-   rbind (hpd.mcmcs, contextHDP) 
    
   hpd.mcmcs $ predictor  <-   gsub ( "context" ,  "" , hpd.mcmcs $ predictor) 
    
    
   single_pred_dat  <-  hpd.mcmcs[ grep ( "risk:|risk$" , 
       hpd.mcmcs $ predictor), ] 
    
   gg_single_pred  <-   ggplot ( data =  single_pred_dat)  +  
        geom_vline ( xintercept =   0 ,  lty =   2 )  +   geom_density_ridges ( aes ( y =  predictor, 
        x =  effect_size),  fill =  cols[ 8 ],  alpha =   0.6 )  +  
        scale_y_discrete ( expand =   c ( 0.01 ,  0 ))  +   scale_x_continuous ( expand =   c ( 0.01 , 
        0 ))  +   labs ( x =   "Effect size" ,  y =   "Interaction" ) 
    
   gg_single_pred        
 
 
   
 
 
 
 
  6.2.2  Foraging efficiency and context 
 
 
 Code 
      agg_dat  <-   aggregate (foraging_efficiency  ~  context, foraging_data, mean) 
    #   
    # ggplot(foraging_data, aes(x = context, y = foraging_efficiency)) +   
    #  geom_violin(fill = cols[7]) +  
    #   geom_point(data = agg_dat, size = 4, color = cols[2]) +  
    #   labs(x = "Context", y = "Foraging efficiency")  
    #   
    
   cols  <-   viridis ( 10 ) 
    
   agg_dat $ n  <-   sapply ( 1  :  nrow (agg_dat),  function (x)  length ( unique (foraging_data $ indiv[foraging_data $ context  ==  agg_dat $ context[x]])))  
   agg_dat $ n.labels  <-   paste ( "n =" , agg_dat $ n) 
    # agg_dat$sensory_input <- factor(agg_dat$sensory_input)  
    # raincoud plot:  
   fill_color  <-   adjustcolor ( "#e85307" ,  0.6 ) 
    
    ggplot (foraging_data,  aes ( x =  context,  y =  foraging_efficiency))  +  
      # add half-violin from {ggdist} package  
     ggdist ::  stat_halfeye ( 
        fill =  fill_color, 
        alpha =   0.5 , 
        # custom bandwidth  
        adjust =  . 5 , 
        # adjust height  
        width =  . 6 , 
        .width =   0 , 
        # move geom to the cright  
        justification =   - . 2 , 
        point_colour =   NA  
     )  +  
      geom_boxplot ( fill =  fill_color, 
        width =  . 15 , 
        # remove outliers  
        outlier.shape =   NA   # `outlier.shape = NA` works as well  
     )  +  
      # add justified jitter from the {gghalves} package  
     gghalves ::  geom_half_point ( 
        color =  fill_color, 
        # draw jitter on the left  
        side =   "l" , 
        # control range of jitter  
        range_scale =  . 4 , 
        # add some transparency  
        alpha =  . 5 , 
     )  +     
      ylim ( c ( 0 ,  0.75 ))  +  
      geom_text ( data =  agg_dat,  aes ( y =   rep ( 0.01 ,  2 ),  x =  context,  label =  n.labels),  nudge_x =   0 ,  size =   6 )  +   
       # scale_x_discrete(labels=c("Control" = "Noise control", "Sound vision" = "Sound & vision", "Vision" = "Vision", "Lessen input" = "Lessen input")) +  
      labs ( x =   "Context" ,  y =   "Foraging efficiency" )         
 
 
  Warning: Using the `size` aesthietic with geom_segment was deprecated in ggplot2 3.4.0.
ℹ Please use the `linewidth` aesthetic instead.  
 
 
   
 
 
 
 
  6.2.3  Scatter plots with best fit lines 
 
 
 Code 
      cols  <-   rep (cols[ 7 ],  10 ) 
    
   out  <-   lapply ( names (best_mods),  function (x){ 
      
     mod  <-  best_mods[[x]] 
       
     pred  <-   predict.MCMCglmm (mod,  interval =   "confidence" ) 
      
     rep_dat  <-   cbind (foraging_data[ !  is.na (foraging_data[, x,  drop =   TRUE ]), ], pred) 
      
      ### both data sets in a single plot  
      # ggplot(rep_dat, aes(x = exploration, y = foraging_efficiency, color = context)) +  
      #   geom_ribbon(aes(ymin = lwr, ymax = upr, fill = context), alpha = .1, show.legend = FALSE, lwd = 0) +  
      #     geom_line(aes(y = fit), size = 1) +  
      #   scale_color_manual(values = cols[c(3, 8)]) +  
      #   geom_point(size = 2) +  
      #   labs(x = "log(exploratory behavior)", y = "Foraging efficiency") +  
      #   theme(legend.position = c(0.8, 0.7), legend.background = element_rect("transparent"))  
      
     gg_hi  <-   ggplot (rep_dat[rep_dat $ context  ==   "High risk" , ],  aes ( x =   get (x),  y =  foraging_efficiency,  color =  context))  +  
        geom_ribbon ( aes ( ymin =  lwr,  ymax =  upr,  fill =  context),  alpha =  . 2 ,  lwd =   0 )  +  
          geom_line ( aes ( y =  fit),  size =   1.5 )  +  
        scale_color_manual ( values =  cols[ 3 ])  +  
        geom_point ( size =   3 )  +  
      labs ( x =   paste0 ( "log(" ,  gsub ( "_" ,  " " , x),  ")" ),  y =   "" )  +   
            theme_classic ( base_size =   20 )  +  
        theme ( legend.position =   "none" ,  axis.text.y =   element_blank (),  axis.ticks.y =   element_blank ()) 
      
     gg_lo  <-   ggplot (rep_dat[rep_dat $ context  !=   "High risk" , ],  aes ( x =   get (x),  y =  foraging_efficiency,  color =  context))  +  
        geom_ribbon ( aes ( ymin =  lwr,  ymax =  upr,  fill =  context),  alpha =  . 2 ,  lwd =   0 )  +  
          geom_line ( aes ( y =  fit),  size =   1.5 )  +  
        scale_color_manual ( values =  cols[ 8 ])  +  
        geom_point ( size =   3 )  +  
          labs ( x =   paste0 ( "log(" ,  gsub ( "_" ,  " " , x),  ")" ),  y =   "Foraging efficiency" )  +  
        theme_classic ( base_size =   20 )  +  
        theme ( legend.position=  "none" ,  axis.title.y =   element_blank ()) 
      
      
     return ( list (gg_lo, gg_hi))  
        
   }) 
    
   plot_list  <-   unlist (out,  recursive =   FALSE ) 
    
    
   pg  <-   plot_grid ( plotlist =  plot_list,  ncol =   2 ,  rel_widths =   c ( 1 ,  1 )) 
    
    # title for left low risk  
   t_lo  <-   ggdraw ()  +   
      draw_label ( 
        "Low risk" , 
        fontface =   'bold' , 
        hjust =   0.5 , 
        size =   20  
       ) 
      
    # title for right high risk  
   t_hi  <-   ggdraw ()  +   
      draw_label ( 
        "High risk" , 
        fontface =   'bold' , 
        hjust =   0.5 , 
        size =   20  
     ) 
    
   ptitles  <-   plot_grid (t_lo, t_hi,  ncol =   2 ,  rel_widths =   c ( 1 ,  0.9 )) 
    
   two_colm_plot  <-   plot_grid ( 
     ptitles, pg, 
      ncol =   1 , 
      # rel_heights values control vertical title margins  
      rel_heights =   c ( 0.1 ,  1 ) 
   ) 
    
   t_ylab  <-   ggdraw ()  +   
      draw_label ( 
        "Foraging efficiency" , 
        fontface =   'bold' , 
        hjust =   0.5 , 
        size =   20 , 
        angle =   90  
     ) 
    
    plot_grid ( 
     t_ylab, two_colm_plot, 
      ncol =   2 , 
      # rel_heights values control vertical title margins  
      rel_widths =   c ( 0.05 ,  1 ) 
     )        
 
 
   
 
 
 Code 
       #######         
 
 
 
 
  As expected, foraging efficiency decreases in high risk contexts  
  Higher arousal is associated with higher foraging efficiency when facing higher risks  
  Highly explorative behavior is increases foraging efficiency when facing lower risks but decreases efficiency at higher risks  
  Risk avoidance tend to lower efficiency but does not differ between risk levels  
 
 
 
 
 
 
  6.3  Single global model 
 Alternatively we can run a single global model that contains all personality parameters and their interaction with context. 
  -->
 
 
  6.3.1  Models 
 We tried 3 types of models from all posible models of interactions between ‘context’ and ‘personality’ parameters, as well as the context only model and the null model: 
 
 context, personality parameters and their interaction as predictors. This included models with 1, 2 and 3 interaction terms (all constitute  alternative hypotheses accounting for individual differences ): 
 
  \[foraging\ efficiency \sim context * person.param1 + (1 | indiv)\]  
  \[foraging\ efficiency \sim context * person.param1 +
context * person.param2 + (1 | indiv)\]  
  \[foraging\ efficiency \sim context * person.param1 + context * person.param2 + context * person.param3 + (1 | indiv)\]  
 
  only context as predictor (i.e.  “classical” hypothesis ):  \[foraging\ efficiency \sim context + (1 | indiv)\]   
  Null model with no predictor:  \[foraging\ efficiency \sim 1 + (1 | indiv)\]   
 
 
   --> 
 
 
 Code 
      foraging_subdata  <-  foraging_data[,  c ( "arousal" , 
        "exploration" ,  "risk_avoidance" ,  "indiv" , 
        "foraging_efficiency" ,  "context" )] 
    
   foraging_subdata  <-  foraging_subdata[ complete.cases (foraging_subdata), 
       ] 
    
   itrns  <-   1e+05  
    
   md_null  <-   replicate ( 3 ,  MCMCglmm (foraging_efficiency  ~  
        1 ,  random =   ~ indiv,  data =  foraging_subdata, 
        verbose =   FALSE ,  nitt =  itrns,  start =   list ( QUASI =   FALSE )), 
        simplify =   FALSE ) 
    
   md_all_interactions  <-   replicate ( 3 ,  MCMCglmm (foraging_efficiency  ~  
       context  *  arousal  +  context  *  exploration  +  
           context  *  risk_avoidance,  random =   ~ indiv, 
        data =  foraging_subdata,  verbose =   FALSE , 
        nitt =  itrns,  start =   list ( QUASI =   FALSE )), 
        simplify =   FALSE ) 
    
   md_arousal_exploration  <-   replicate ( 3 ,  MCMCglmm (foraging_efficiency  ~  
       context  *  arousal  +  context  *  exploration, 
        random =   ~ indiv,  data =  foraging_subdata, 
        verbose =   FALSE ,  nitt =  itrns,  start =   list ( QUASI =   FALSE )), 
        simplify =   FALSE ) 
    
   md_arousal_risk_avoidance  <-   replicate ( 3 ,  MCMCglmm (foraging_efficiency  ~  
       context  *  arousal  +  context  *  risk_avoidance, 
        random =   ~ indiv,  data =  foraging_subdata, 
        verbose =   FALSE ,  nitt =  itrns,  start =   list ( QUASI =   FALSE )), 
        simplify =   FALSE ) 
    
   md_risk_avoidance_exploration  <-   replicate ( 3 , 
        MCMCglmm (foraging_efficiency  ~  context  *  risk_avoidance  +  
           context  *  exploration,  random =   ~ indiv, 
            data =  foraging_subdata,  verbose =   FALSE , 
            nitt =  itrns,  start =   list ( QUASI =   FALSE )), 
        simplify =   FALSE ) 
    
    # single interaction models  
   md_arousal  <-   replicate ( 3 ,  MCMCglmm (foraging_efficiency  ~  
       context  *  arousal,  random =   ~ indiv,  data =  foraging_subdata, 
        verbose =   FALSE ,  nitt =  itrns,  start =   list ( QUASI =   FALSE )), 
        simplify =   FALSE ) 
    
   md_risk_avoidance  <-   replicate ( 3 ,  MCMCglmm (foraging_efficiency  ~  
       context  *  risk_avoidance,  random =   ~ indiv, 
        data =  foraging_subdata,  verbose =   FALSE , 
        nitt =  itrns,  start =   list ( QUASI =   FALSE )), 
        simplify =   FALSE ) 
    
   md_exploration  <-   replicate ( 3 ,  MCMCglmm (foraging_efficiency  ~  
       context  *  exploration,  random =   ~ indiv,  data =  foraging_subdata, 
        verbose =   FALSE ,  nitt =  itrns,  start =   list ( QUASI =   FALSE )), 
        simplify =   FALSE ) 
    
   md_context  <-   replicate ( 3 ,  MCMCglmm (foraging_efficiency  ~  
       context,  random =   ~ indiv,  data =  foraging_subdata, 
        verbose =   FALSE ,  nitt =  itrns,  start =   list ( QUASI =   FALSE )), 
        simplify =   FALSE ) 
    
    # put together the first models  
   msDIC  <-   model.sel (md_null[[ 1 ]], md_all_interactions[[ 1 ]], 
       md_arousal_exploration[[ 1 ]], md_arousal_risk_avoidance[[ 1 ]], 
       md_risk_avoidance_exploration[[ 1 ]], md_risk_avoidance[[ 1 ]], 
       md_arousal[[ 1 ]], md_exploration[[ 1 ]], md_context[[ 1 ]], 
        rank =   "DIC" ) 
    
    # rename delta and weight  
    names (msDIC)[ names (msDIC)  %in%   c ( "delta" ,  "weight" )]  <-   paste0 ( "DIC." , 
        c ( "delta" ,  "weight" )) 
    
   msAIC  <-   model.sel (md_null[[ 1 ]], md_all_interactions[[ 1 ]], 
       md_arousal_exploration[[ 1 ]], md_arousal_risk_avoidance[[ 1 ]], 
       md_risk_avoidance_exploration[[ 1 ]], md_risk_avoidance[[ 1 ]], 
       md_arousal[[ 1 ]], md_exploration[[ 1 ]], md_context[[ 1 ]], 
        rank =   "AIC" ) 
    
    # rename delta and weight  
    names (msAIC)[ names (msAIC)  %in%   c ( "delta" ,  "weight" )]  <-   paste0 ( "AIC." , 
        c ( "delta" ,  "weight" )) 
    
   ms  <-   cbind (msDIC, msAIC[,  c ( "AIC" ,  "AIC.delta" , 
        "AIC.weight" )]) 
    
    # rename rows so they match predictor names  
    rownames (ms)  <-   gsub ( "[[1]]" ,  "" ,  rownames (ms), 
        fixed =   TRUE ) 
    
    # save models in a list  
   res  <-   list ( model.tab =  ms,  md_all_interactions =  md_all_interactions, 
        md_arousal_exploration =  md_arousal_exploration, 
        md_arousal_risk_avoidance =  md_arousal_risk_avoidance, 
        md_risk_avoidance_exploration =  md_risk_avoidance_exploration, 
        md_risk_avoidance =  md_risk_avoidance,  md_arousal =  md_arousal, 
        md_exploration =  md_exploration,  md_context =  md_context, 
        md_null =  md_null) 
    
    saveRDS (res,  "model_selection_all_parameters_foraging_efficiency.RDS" )        
 
 
 
 
  6.3.2  Model selection 
 
 
 Code 
      mcmc_output  <-   readRDS ( "./output/model_selection_all_parameters_foraging_efficiency.RDS" ) 
    
    # make a data frame with all results  
   mod.sel.tab  <-   data.frame ( response =   "Foraging efficiency" , 
        predictors =   rownames (mcmc_output[[ 1 ]]),  as.data.frame (mcmc_output[[ 1 ]]), 
        stringsAsFactors =   FALSE ) 
    
    # rename predictors for table  
    rownames (mod.sel.tab)  <-   gsub ( "md_" ,  "" ,  rownames (mod.sel.tab)) 
    
   mod.sel.tab $ DIC.delta  <-   round (mod.sel.tab $ DIC.delta, 
        2 ) 
   mod.sel.tab $ DIC.weight  <-   round (mod.sel.tab $ DIC.weight, 
        2 ) 
   mod.sel.tab $ AIC.delta  <-   round (mod.sel.tab $ AIC.delta, 
        2 ) 
   mod.sel.tab $ AIC.weight  <-   round (mod.sel.tab $ AIC.weight, 
        2 ) 
    
    options ( knitr.kable.NA =   "" ) 
    
   df1  <-  knitr ::  kable (mod.sel.tab[,  c ( "response" , 
        "predictors" ,  "df" ,  "DIC" ,  "DIC.delta" ,  "DIC.weight" , 
        "AIC" ,  "AIC.delta" ,  "AIC.weight" )],  row.names =   FALSE , 
        escape =   FALSE ,  format =   "html" ) 
    
   df1  <-   row_spec (df1,  which (mod.sel.tab $ DIC.delta  ==  
        0 ),  background =   adjustcolor (cols[ 9 ],  alpha.f =   0.3 )) 
    
    kable_styling (df1,  bootstrap_options =   c ( "striped" , 
        "hover" ,  "condensed" ,  "responsive" ),  full_width =   FALSE , 
        font_size =   11 )        
 
 

 
  
   
     response  
     predictors  
     df  
     DIC  
     DIC.delta  
     DIC.weight  
     AIC  
     AIC.delta  
     AIC.weight  
   
  
 
   
     Foraging efficiency  
     md_all_interactions  
     10  
     -400.0909  
     0.00  
     1  
     -396.3073  
     0.00  
     0.99  
   
   
     Foraging efficiency  
     md_arousal_exploration  
     8  
     -388.2385  
     11.85  
     0  
     -386.2831  
     10.02  
     0.01  
   
   
     Foraging efficiency  
     md_arousal_risk_avoidance  
     8  
     -378.9807  
     21.11  
     0  
     -376.8184  
     19.49  
     0.00  
   
   
     Foraging efficiency  
     md_arousal  
     6  
     -363.3410  
     36.75  
     0  
     -363.2509  
     33.06  
     0.00  
   
   
     Foraging efficiency  
     md_risk_avoidance_exploration  
     8  
     -350.1568  
     49.93  
     0  
     -348.8140  
     47.49  
     0.00  
   
   
     Foraging efficiency  
     md_exploration  
     6  
     -345.7716  
     54.32  
     0  
     -346.4065  
     49.90  
     0.00  
   
   
     Foraging efficiency  
     md_risk_avoidance  
     6  
     -315.2258  
     84.87  
     0  
     -315.0929  
     81.21  
     0.00  
   
   
     Foraging efficiency  
     md_context  
     4  
     -308.6036  
     91.49  
     0  
     -310.7995  
     85.51  
     0.00  
   
   
     Foraging efficiency  
     md_null  
     3  
     -296.3098  
     103.78  
     0  
     -299.8347  
     96.47  
     0.00  
   
 
 

 
 
 
 
 Best model includes all interactions 
 
 
 
 
  6.3.3  Effect sizes for best model 
 
 
 Code 
       # select best models based on BIC  
   best_mod  <-  mcmc_output[[ 2 ]] 
    
    # fixed effects  
   fe  <-   summary (best_mod[[ 1 ]]) $ solutions 
    
    # Confidence intervals  
   ci  <-   HPDinterval (best_mod[[ 1 ]] $ Sol) 
    
    # observations used  
   obs  <-  foraging_data[ complete.cases (foraging_data[,  c ( "arousal" ,  "exploration" ,  "risk_avoidance" ,  "indiv" ,  "foraging_efficiency" ,  "context" )]), ] 
      
    # put results together in a data frame  
   effect_size_single_model  <-   data.frame ( 
      stringsAsFactors =   FALSE ,  
      # response variable name  
      response =   "foraging effiency" ,  
      # predictor name  
      predictor =   rownames (ci)[ 2  :  nrow (ci)],  
      effect_size =  fe[ -  1 ,  "post.mean" ],  
      # lower confident interval  
      CI_2.5 =  ci[ 2  :  nrow (ci),  1 ],  
      # upper confident interval  
      CI_97.5 =  ci[ 2  :  nrow (ci),  2 ],  
      # p value  
      pMCMC  =  fe[ -  1 ,  "pMCMC" ],  
      #intercept  
      intercept =  fe[ 1 ,  "post.mean" ], 
      # number of individuals  
      n.indv =   length ( unique (obs $ indiv)),  
      # number of observations  
      n.obs =   nrow (obs) 
   ) 
      
    rownames (effect_size_single_model)  <-   1  :  nrow (effect_size_single_model) 
    
    
   md  <-  effect_size_single_model[,  !  grepl ( "mean|sd" ,  names (effect_size_single_model))] 
    
   md $ CI_2 .5   <-   round (md $ CI_2 .5 ,  4 ) 
   md $ CI_97 .5   <-   round (md $ CI_97 .5 ,  4 ) 
    
    # get the ones that do not overlap with 0  
   mltp  <-  md $ CI_2 .5   *  md $ CI_97 .5  
    
   md $ CI_2 .5   <-   ifelse (mltp  >   0 ,  cell_spec (md $ CI_2 .5 ,  "html" ,  color =  "white" ,  background =  cols[ 7 ],  bold =  T,   font_size =   12 ),   cell_spec (md $ CI_2 .5 ,  "html" )) 
    
   md $ CI_97 .5   <-   ifelse (mltp  >   0 ,  cell_spec (md $ CI_97 .5 ,  "html" ,  color =  "white" ,  background =  cols[ 7 ],  bold =  T,   font_size =   12 ),   cell_spec (md $ CI_97 .5 ,  "html" )) 
    
   df1  <-  knitr ::  kable (md,  row.names =   FALSE ,  escape =   FALSE ,  format =   "html" ,  digits =   c ( 4 )) 
    
   df1  <-   row_spec (df1,  which (mltp  >   0 ),  background =   adjustcolor (cols[ 9 ],  alpha.f =   0.3 )) 
      
    kable_styling (df1,  bootstrap_options =   c ( "striped" ,  "hover" ,  "condensed" ,  "responsive" ),  full_width =   FALSE ,  font_size =   12 )        
 
 

 
  
   
     response  
     predictor  
     effect_size  
     CI_2.5  
     CI_97.5  
     pMCMC  
     intercept  
     n.indv  
     n.obs  
   
  
 
   
     foraging effiency  
     contextHigh risk  
     -0.1409  
      -0.2732   
      -0.0132   
     0.0322  
     0.4548  
     11  
     192  
   
   
     foraging effiency  
     arousal  
     0.0684  
      0.0275   
      0.1083   
     0.0006  
     0.4548  
     11  
     192  
   
   
     foraging effiency  
     exploration  
     0.3686  
      0.1244   
      0.6167   
     0.0023  
     0.4548  
     11  
     192  
   
   
     foraging effiency  
     risk_avoidance  
     -0.0327  
      -0.0663   
      0.0023   
     0.0641  
     0.4548  
     11  
     192  
   
   
     foraging effiency  
     contextHigh risk:arousal  
     0.2445  
      0.1541   
      0.3436   
     0.0001  
     0.4548  
     11  
     192  
   
   
     foraging effiency  
     contextHigh risk:exploration  
     -0.8355  
      -1.1641   
      -0.4925   
     0.0001  
     0.4548  
     11  
     192  
   
   
     foraging effiency  
     contextHigh risk:risk_avoidance  
     -0.0270  
      -0.0793   
      0.021   
     0.2918  
     0.4548  
     11  
     192  
   
 
 

 
 
 
 
  Similar to single predictor models  
  Risk avoidance doesn’t affect foraging efficiency  
 
 
 
 
  6.3.4  Effect sizes (on foraging efficiency) for interaction terms 
 
 
 Code 
       # effect_size_single_model$predictor <-  
    # gsub('context', '',  
    # effect_size_single_model$predictor)  
    # ggplot(effect_size_single_model[grep('risk:',  
    # effect_size_single_model$predictor), ],  
    # aes(x = predictor, y = effect_size)) +  
    # geom_hline(yintercept = 0, lty = 2) +  
    # geom_point(col = cols[7], size = 5) +  
    # geom_errorbar(aes(ymin=CI_2.5,  
    # ymax=CI_97.5), width= 0, col = cols[7],  
    # size = 2) + coord_flip()  
    
   hpd.mcmcs  <-   HPD_mcmc (best_mod[[ 1 ]] $ Sol) 
    
    # remove other context predictors  
   hpd.mcmcs  <-  hpd.mcmcs[hpd.mcmcs $ predictor  !=  
        "contextHigh risk" , ] 
    
    # add context  
   hpd.mcmcs.context  <-   HPD_mcmc (mcmc_output $ md_context[[ 1 ]] $ Sol) 
    
   hpd.mcmcs  <-   rbind (hpd.mcmcs, hpd.mcmcs.context) 
    
    
   hpd.mcmcs $ predictor  <-   gsub ( "context" ,  "" , hpd.mcmcs $ predictor) 
    
   effect_size_single_model $ predictor  <-   gsub ( "context" , 
        "" , effect_size_single_model $ predictor) 
    
   single_mod_dat  <-  hpd.mcmcs[ grep ( "risk:|risk$" , 
       hpd.mcmcs $ predictor), ] 
    
   gg_single_mod  <-   ggplot ( data =  single_mod_dat)  +  
        geom_vline ( xintercept =   0 ,  lty =   2 )  +   geom_density_ridges ( aes ( y =  predictor, 
        x =  effect_size),  fill =  cols[ 8 ],  alpha =   0.6 )  +  
        scale_y_discrete ( expand =   c ( 0.01 ,  0 ))  +   scale_x_continuous ( expand =   c ( 0.01 , 
        0 ))  +   labs ( x =   "Effect size" ,  y =   "Interaction" ) 
    
   gg_single_mod        
 
 
   
 
 
 
 Similar to single predictor models: 
 
  Foraging effiency decreases in high risk contexts  
  Higher arousal is associated with higher foraging efficiency when facing higher risks  
  Higher exploration is associated with lower foraging efficiency when facing higher risks  
  Risk avoidance does not affect significantly  
 
 
 Look at estimates from single predictor models and global model: 
 
 
 Code 
      single_pred_dat $ models  <-   "single predictor"  
   single_mod_dat $ models  <-   "single model"  
    
   mods_dat  <-   rbind (single_pred_dat, single_mod_dat) 
    
    ggplot ( data =  mods_dat[mods_dat $ predictor  !=   "High risk" , 
       ])  +   geom_vline ( xintercept =   0 ,  lty =   2 )  +  
        geom_density_ridges ( aes ( y =  predictor,  x =  effect_size, 
            fill =  models),  alpha =   0.6 )  +   scale_fill_viridis_d ( begin =   0.4 , 
        end =   0.9 )  +   scale_y_discrete ( expand =   c ( 0.01 , 
        0 ))  +   scale_x_continuous ( expand =   c ( 0.01 , 
        0 ))  +   theme ( legend.position =   c ( 0.36 ,  0.9 ))  +  
        labs ( x =   "Effect size" ,  y =   "Interaction" )        
 
 
   
 
 
 

 
 
 
 Results are consistent despite of the statistical approach 
 
 
 

 
 
 
 
  6.4  Diagnostic stats and plots on MCMCglmm models 
 
  6.4.1  Single parameter models 
 
 
 Code 
       # read skipping model selection table  
   mcmc_single_param  <-   readRDS ( "./output/model_selection_predict_foraging_efficiency.RDS" ) 
    
    for  (w  in   1  :  length (mcmc_single_param)) { 
        print ( paste ( "Predictor:" ,  names (mcmc_single_param)[w])) 
    
       mods  <-  mcmc_single_param[[w]][ -  1 ] 
    
        for  (x  in   1  :  length (mods)) { 
    
            print ( names (mods)[x]) 
    
           X  <-  mods[[x]] 
            plot_repl_mcmc_models (X,  begin =   0.4 ) 
       } 
   }        
 
 
  [1] "Predictor: arousal"
[1] "md_only_context"  
 
 
   
 
 
   
 
 
   
 
 
   
 
 
   
 
 
   
 
 
   
 
 
   
 
 
   
 
 
   
 
 
  [1] "md_only_parameter"  
 
 
   
 
 
   
 
 
   
 
 
   
 
 
   
 
 
   
 
 
   
 
 
   
 
 
   
 
 
   
 
 
  [1] "md_interation"  
 
 
   
 
 
   
 
 
   
 
 
   
 
 
   
 
 
   
 
 
   
 
 
   
 
 
   
 
 
   
 
 
   
 
 
   
 
 
   
 
 
   
 
 
   
 
 
   
 
 
   
 
 
   
 
 
   
 
 
  [1] "md_null"  
 
 
   
 
 
   
 
 
   
 
 
   
 
 
   
 
 
   
 
 
  [1] "Predictor: exploration"
[1] "md_only_context"  
 
 
   
 
 
   
 
 
   
 
 
   
 
 
   
 
 
   
 
 
   
 
 
   
 
 
   
 
 
   
 
 
  [1] "md_only_parameter"  
 
 
   
 
 
   
 
 
   
 
 
   
 
 
   
 
 
   
 
 
   
 
 
   
 
 
   
 
 
   
 
 
  [1] "md_interation"  
 
 
   
 
 
   
 
 
   
 
 
   
 
 
   
 
 
   
 
 
   
 
 
   
 
 
   
 
 
   
 
 
   
 
 
   
 
 
   
 
 
   
 
 
   
 
 
   
 
 
   
 
 
   
 
 
   
 
 
  [1] "md_null"  
 
 
   
 
 
   
 
 
   
 
 
   
 
 
   
 
 
   
 
 
  [1] "Predictor: risk_avoidance"
[1] "md_only_context"  
 
 
   
 
 
   
 
 
   
 
 
   
 
 
   
 
 
   
 
 
   
 
 
   
 
 
   
 
 
   
 
 
  [1] "md_only_parameter"  
 
 
   
 
 
   
 
 
   
 
 
   
 
 
   
 
 
   
 
 
   
 
 
   
 
 
   
 
 
   
 
 
  [1] "md_interation"  
 
 
   
 
 
   
 
 
   
 
 
   
 
 
   
 
 
   
 
 
   
 
 
   
 
 
   
 
 
   
 
 
   
 
 
   
 
 
   
 
 
   
 
 
   
 
 
   
 
 
   
 
 
   
 
 
   
 
 
  [1] "md_null"  
 
 
   
 
 
   
 
 
   
 
 
   
 
 
   
 
 
   
 
 
 
 
  6.4.2  Global model 
 
 
 Code 
       # read skipping model selection table  
   mcmc_all_param  <-   readRDS ( "./output/model_selection_all_parameters_foraging_efficiency.RDS" )[ -  1 ] 
    
    for  (x  in   1  :  length (mcmc_all_param)) { 
    
        print ( names (mcmc_all_param)[x]) 
    
       X  <-  mcmc_all_param[[x]] 
        plot_repl_mcmc_models (X,  begin =   0.4 ) 
   }        
 
 
  [1] "md_all_interactions"  
 
 
   
 
 
   
 
 
   
 
 
   
 
 
   
 
 
   
 
 
   
 
 
   
 
 
   
 
 
   
 
 
   
 
 
   
 
 
   
 
 
   
 
 
   
 
 
   
 
 
   
 
 
   
 
 
   
 
 
   
 
 
   
 
 
   
 
 
   
 
 
   
 
 
   
 
 
   
 
 
   
 
 
   
 
 
   
 
 
   
 
 
   
 
 
   
 
 
   
 
 
   
 
 
   
 
 
   
 
 
   
 
 
  [1] "md_arousal_exploration"  
 
 
   
 
 
   
 
 
   
 
 
   
 
 
   
 
 
   
 
 
   
 
 
   
 
 
   
 
 
   
 
 
   
 
 
   
 
 
   
 
 
   
 
 
   
 
 
   
 
 
   
 
 
   
 
 
   
 
 
   
 
 
   
 
 
   
 
 
   
 
 
   
 
 
   
 
 
   
 
 
   
 
 
   
 
 
  [1] "md_arousal_risk_avoidance"  
 
 
   
 
 
   
 
 
   
 
 
   
 
 
   
 
 
   
 
 
   
 
 
   
 
 
   
 
 
   
 
 
   
 
 
   
 
 
   
 
 
   
 
 
   
 
 
   
 
 
   
 
 
   
 
 
   
 
 
   
 
 
   
 
 
   
 
 
   
 
 
   
 
 
   
 
 
   
 
 
   
 
 
   
 
 
  [1] "md_risk_avoidance_exploration"  
 
 
   
 
 
   
 
 
   
 
 
   
 
 
   
 
 
   
 
 
   
 
 
   
 
 
   
 
 
   
 
 
   
 
 
   
 
 
   
 
 
   
 
 
   
 
 
   
 
 
   
 
 
   
 
 
   
 
 
   
 
 
   
 
 
   
 
 
   
 
 
   
 
 
   
 
 
   
 
 
   
 
 
   
 
 
  [1] "md_risk_avoidance"  
 
 
   
 
 
   
 
 
   
 
 
   
 
 
   
 
 
   
 
 
   
 
 
   
 
 
   
 
 
   
 
 
   
 
 
   
 
 
   
 
 
   
 
 
   
 
 
   
 
 
   
 
 
   
 
 
   
 
 
  [1] "md_arousal"  
 
 
   
 
 
   
 
 
   
 
 
   
 
 
   
 
 
   
 
 
   
 
 
   
 
 
   
 
 
   
 
 
   
 
 
   
 
 
   
 
 
   
 
 
   
 
 
   
 
 
   
 
 
   
 
 
   
 
 
  [1] "md_exploration"  
 
 
   
 
 
   
 
 
   
 
 
   
 
 
   
 
 
   
 
 
   
 
 
   
 
 
   
 
 
   
 
 
   
 
 
   
 
 
   
 
 
   
 
 
   
 
 
   
 
 
   
 
 
   
 
 
   
 
 
  [1] "md_context"  
 
 
   
 
 
   
 
 
   
 
 
   
 
 
   
 
 
   
 
 
   
 
 
   
 
 
   
 
 
   
 
 
  [1] "md_null"  
 
 
   
 
 
   
 
 
   
 
 
   
 
 
   
 
 
   
 
 
 
  Session information  
 
 
  R version 4.1.0 (2021-05-18)
Platform: x86_64-pc-linux-gnu (64-bit)
Running under: Ubuntu 20.04.2 LTS

Matrix products: default
BLAS:   /usr/lib/x86_64-linux-gnu/atlas/libblas.so.3.10.3
LAPACK: /usr/lib/x86_64-linux-gnu/atlas/liblapack.so.3.10.3

locale:
 [1] LC_CTYPE=pt_BR.UTF-8       LC_NUMERIC=C              
 [3] LC_TIME=es_CR.UTF-8        LC_COLLATE=pt_BR.UTF-8    
 [5] LC_MONETARY=es_CR.UTF-8    LC_MESSAGES=pt_BR.UTF-8   
 [7] LC_PAPER=es_CR.UTF-8       LC_NAME=C                 
 [9] LC_ADDRESS=C               LC_TELEPHONE=C            
[11] LC_MEASUREMENT=es_CR.UTF-8 LC_IDENTIFICATION=C       

attached base packages:
[1] grid      parallel  stats     graphics  grDevices utils     datasets 
[8] methods   base     

other attached packages:
 [1] gridExtra_2.3     ggplotify_0.1.0   cowplot_1.1.1     ggridges_0.5.4   
 [5] kableExtra_1.3.4  MuMIn_1.43.17     pbapply_1.7-0     rptR_0.9.22      
 [9] corrplot_0.90     MCMCglmm_2.32     ape_5.6-2         coda_0.19-4      
[13] ggpubr_0.4.0      smatr_3.4-8       lmerTest_3.1-3    lme4_1.1-27.1    
[17] Matrix_1.5-1      forcats_0.5.1     stringr_1.5.0     dplyr_1.0.10     
[21] purrr_1.0.0       readr_2.1.3       tidyr_1.1.3       tibble_3.1.8     
[25] tidyverse_1.3.1   ggplot2_3.4.0     readxl_1.3.1      viridis_0.6.2    
[29] viridisLite_0.4.1

loaded via a namespace (and not attached):
 [1] cubature_2.0.4.2     minqa_1.2.4          colorspace_2.0-3    
 [4] ggsignif_0.6.2       ellipsis_0.3.2       rio_0.5.27          
 [7] corpcor_1.6.9        fs_1.6.0             xaringanExtra_0.7.0 
[10] rstudioapi_0.14      farver_2.1.1         remotes_2.4.2       
[13] fansi_1.0.3          lubridate_1.7.10     xml2_1.3.3          
[16] splines_4.1.0        knitr_1.42           jsonlite_1.8.4      
[19] nloptr_1.2.2.2       packrat_0.9.0        broom_0.7.8         
[22] dbplyr_2.1.1         ggdist_3.2.0         compiler_4.1.0      
[25] httr_1.4.4           backports_1.4.1      assertthat_0.2.1    
[28] fastmap_1.1.1        cli_3.6.1            formatR_1.11        
[31] htmltools_0.5.5      tools_4.1.0          gtable_0.3.1        
[34] glue_1.6.2           Rcpp_1.0.10          carData_3.0-4       
[37] cellranger_1.1.0     vctrs_0.6.2          svglite_2.1.0       
[40] nlme_3.1-152         gghalves_0.1.3       tensorA_0.36.2      
[43] xfun_0.39            openxlsx_4.2.4       rvest_1.0.3         
[46] lifecycle_1.0.3      rstatix_0.7.0        MASS_7.3-54         
[49] scales_1.2.1         hms_1.1.2            yaml_2.3.7          
[52] curl_4.3.3           yulab.utils_0.0.5    stringi_1.7.12      
[55] boot_1.3-28          zip_2.2.2            rlang_1.1.1         
[58] pkgconfig_2.0.3      systemfonts_1.0.4    distributional_0.3.1
[61] evaluate_0.21        lattice_0.20-44      labeling_0.4.2      
[64] htmlwidgets_1.5.4    tidyselect_1.2.0     magrittr_2.0.3      
[67] R6_2.5.1             generics_0.1.3       DBI_1.1.3           
[70] pillar_1.8.1         haven_2.4.1          foreign_0.8-81      
[73] withr_2.5.0          abind_1.4-5          modelr_0.1.8        
[76] crayon_1.5.2         car_3.0-11           utf8_1.2.2          
[79] tzdb_0.3.0           rmarkdown_2.20       sketchy_1.0.2       
[82] data.table_1.14.0    reprex_2.0.0         digest_0.6.31       
[85] webshot_0.5.4        numDeriv_2016.8-1.1  gridGraphics_0.5-1  
[88] stats4_4.1.0         munsell_0.5.0         
 
 
 

 
 
 

 
 
      Source Code      
       ---  
    title:   LBH foraging efficiency  
    subtitle:   Statistical analysis  
    author:   <a href="https://maRce10.github.io">Marcelo Araya-Salas, PhD</a>  
    date:   "`r Sys.Date()`"  
    toc:   true  
    toc-depth:   2  
    toc-location:   left  
    number-sections:   true  
    highlight-style:   pygments  
    format:  
      html:  
        df-print: kable  
        code-fold: show  
        code-tools: true  
        css: qmd.css  
    ---  
    
    
    <!-- this code add line numbers to code blocks -->  
    <!-- only works when code folding is not used in yaml (code_folding: show) -->  
    
    ```{=html}  
    <style>  
    body  
      { counter-reset: source-line 0; }  
    pre.numberSource code  
      { counter-reset: none; }  
    </style>  
    ```  
    
    
    ```{r setup style, echo = FALSE, message = FALSE, warning=FALSE}  
    
    # options to customize chunk outputs  
   knitr :: opts_chunk $  set ( 
      class.source =   "numberLines lineAnchors" ,  # for code line numbers  
      tidy.opts =   list ( width.cutoff =   45 ),  
      tidy =   TRUE , 
      message =   FALSE  
    ) 
    
    
   knitr :: opts_knit $  set ( root.dir =   ".." ) 
    
    
    
    ```  
    
    
    <!-- skyblue box -->  
    <div   class  =  "alert alert-info"  >  
    
   Statistical analysis for the paper: 
    
    -  Wojczulanis-Jakubas, K.; Araya-Salas, M. Foraging, Fear and Behavioral Variation in a Traplining Hummingbird. Animals 2023, 13, x. https://doi.org/10.3390/xxxxx 
    
    </div>  
    
    &nbsp;   
    
    ```{r add link to github repo, echo = FALSE, results='asis'}  
    
    # print link to github repo if any  
    if  ( file.exists ( "./.git/config" )){ 
     config  <-   readLines ( "./.git/config" ) 
     url  <-   grep ( "url" ,  config,  value =   TRUE ) 
     url  <-   gsub ( "  \\  turl = |.git$" ,  "" , url) 
      cat ( "  \n  Source code and data found at [" , url,  "](" , url,  ")" ,  sep =   "" ) 
     } 
    
    ```  
    
    
    #  Load packages  
    ```{r packages, message=FALSE, warning = FALSE, echo = TRUE, eval = TRUE}  
    
    ## add 'developer/' to packages to be installed from github  
   x  <-   c ( 
      "viridis" , 
      "readxl" , 
      "ggplot2" , 
      "tidyverse" , 
      "lmerTest" , 
      "lme4" , 
      "smatr" , 
      "ggpubr" , 
      "MCMCglmm" , 
      "corrplot" , 
      "rptR" , 
      "pbapply" , 
      "MuMIn" , 
      "parallel" , 
      "kableExtra" , 
      "ggridges" , 
      "cowplot" , 
      "ggplotify" , 
      "gridExtra" , 
      "grid"  
     ) 
      
   sketchy ::  load_packages (x) 
    
    
    ```  
    
    # Load data and set parameters  
    ```{r functions and parameters, message = FALSE, warning = FALSE, echo = TRUE, eval = TRUE}  
    
   cols  <-   viridis ( 10 ,  alpha =   0.6 ) 
    
    # function to get posterior estimates within the HPD interval  
   HPD_mcmc  <-   function (y,  long =   TRUE ) { 
      
     out  <-   lapply ( 1  :  ncol (y),  function (x){ 
        # calculate hpd  
       hpd  <-   HPDinterval (y[, x]) 
        
        # get sol as vector  
       vctr  <-  y[, x] 
        
        # clip vector to hpd range  
       hpdmcmc  <-  vctr[vctr  >  hpd[ 1 ]  &  vctr  <  hpd[ 2 ]] 
      
        return (hpdmcmc)   
     }) 
      
      # get them together  
     hpd.mcmcs  <-   do.call (cbind, out)   
      
      # change colnames  
      colnames (hpd.mcmcs)  <-   colnames (y) 
    
      # put it in long format  
      if  (long){ 
     est.df  <-   lapply ( 1  :  ncol (hpd.mcmcs),  function (x){ 
        
      data.frame ( predictor =   colnames (hpd.mcmcs)[x],  effect_size =  hpd.mcmcs[, x],  stringsAsFactors =   FALSE ) 
     }) 
      
      # get them together  
     hpd.mcmcs  <-   do.call (rbind, est.df)   
     } 
      
      return (hpd.mcmcs) 
     } 
    
    # color for corrplot  
   col.crrplt  <-   colorRampPalette ( c (cols[ 1  :  2 ],  rep ( "white" ,  1 ), cols[ 6  :  7 ]))( 100 ) 
    
    
    # plot diagonostic stuff for mcmcglmmm models  
   plot_repl_mcmc_models  <-   function (X,  pal =  viridis,  begin =   0.1 ,  end =   1 ) { 
        
      # extract mcmc chains  
     sol_l  <-   lapply (X,  "[[" ,  'Sol' ) 
    
      # put them in a single matrix  
     sol_mat  <-   do.call (cbind, sol_l) 
      
      colnames (sol_mat)  <-   paste0 ( colnames (sol_mat),  " repl" ,  rep ( 1  :  length (X),  each =   ncol (sol_l[[ 1 ]]))) 
      
       sol_mat  <-  sol_mat[,  order ( colnames (sol_mat))] 
      
      # add class attributes of MCMC chains  
      class (sol_mat)  <-   "mcmc"  
      attr (sol_mat,  "mcpar" )  <-   attr (X[[ 1 ]] $ Sol,  "mcpar" )  
    
      # extract each column as a mcmc matrix  
     mcmcs  <-   lapply ( seq_len ( ncol (sol_mat)),  function (x) sol_mat[, x,  drop =   FALSE ]) 
    
     cols  <-   pal ( length (mcmcs),  alpha =   0.7 ,  begin =  begin,  end =  end) 
       
      # test colors  
      # plot(1:length(cols), col = cols, pch = 20, cex =4)  
      
      for (y  in   1  :  length (mcmcs)){ 
    
        # trace and density  
        plot (mcmcs[[y]],  col =  cols[y]) 
    
     }  
      
      # autocorrelation    
      par ( mfrow =   c ( 1 ,  2 )) 
      for (y  in   1  :  length (mcmcs)){ 
    
        autocorr.plot (mcmcs[[y]],  col =  cols[y],  lwd =   4 ,  ask =   FALSE ,  auto.layout =   FALSE ) 
         }  
    
       par ( mfrow =   c ( 1 ,  1 )) 
      ## add global plots and gelman test  
      # gelman_diagnostic  
     gel_diag  <-   as.data.frame ( gelman.diag ( mcmc.list (sol_l)) $ psrf) 
      
      # add estimate as column  
     gel_diag $ estimate  <-   rownames (gel_diag)  
      
      # reorder columns  
     gel_diag  <-  gel_diag[,  c ( 3 ,  1 ,  2 )] 
    
      # plot table  
      grid.newpage () 
      grid.draw ( tableGrob (gel_diag,  rows =   NULL ,  theme=  ttheme_default ( base_size =   25 ))) 
   } 
    
    
    ##### chunk output stuff  
   knitr :: opts_chunk $  set ( dpi =   58 ,  fig.width =   12 ,  fig.height =   8 )  
    
    # ggplot2 theme  
    theme_set ( theme_classic ( base_size =   30 ,  base_family =   "Arial" )) 
    
    
    ## data  
   foraging_data  <-  ff  <-   read_excel ( "./data/raw/ff.xlsx" ) 
    
    names (foraging_data)[ names (foraging_data)  ==   "ID.." ]  <-   "indiv"  
    
    ```  
    
    ```{r kasia code, message = FALSE, warning = FALSE, echo = FALSE, eval = FALSE}  
    
    # overall pattern - ctrl vs treat  
    
    # plot  
   forplot  <-   ggplot ( data =  ff,  aes ( x =  treat,  y =  for_eff))  +  
      geom_boxplot ()  +  
      labs ( x =   "" ,  y =    "Foraging efficiency" )  #+  
      # theme_bw();  
     forplot 
    
    # test  
   foreff_model  <-   lmer (for_eff  ~  treat   +  ( 1   |  ID..),  data =  ff,  REML =   FALSE );  summary (foreff_model) 
    
    # significance of the random factor (birdID)  
   foreff_model_fix  <-   lm (for_eff  ~  treat,  data =  ff)  
    anova (foreff_model, foreff_model_fix) 
    
    # plot for individuals (means per ind)  
   foreff_long  <-  ff  %>%   group_by (ID.., treat)  %>%   
      summarise  ( mfor_eff =   mean (for_eff))  %>%   
      spread ( key =  treat,  value =  mfor_eff,  NA )  %>%   
      remove_missing ()  %>%   
      gather ( key =  treat,  value =  mfor_eff,  - ID..) 
    
   indplot  <-   ggplot ( data =  foreff_long,  aes ( x =  treat,  y =  mfor_eff))  +  
      geom_point ( size =   2 )  +   geom_line ( aes ( group =   as.factor (ID..)))  +   #theme_bw() +  
      labs ( x =   "" ,  y =   "Average foraging efficiency" );indplot 
    
    # ggarrange(forplot, indplot, labels = c("A", "B"),  
    #           ncol = 2, nrow = 1)  
    # ggsave(filename = "forplots.tiff", plot = last_plot(),dpi = 300)  
    
    
    # Arousal ---------------------------------------------------  
    
    # test  
   model.arous  <-   lmer (for_eff  ~  treat  *  mov_feroc_stand  +  ( 1   |  ID..),  
                        data =  ff,  REML =   FALSE );  summary (model.arous) 
    
    # significance of the random factor  
   model.arous_fix  <-   lm (for_eff  ~  treat  *  mov_feroc_stand,  data =  ff)  
    anova (model.arous, model.arous_fix) 
    
    # plot  
   arousalplot  <-   ggplot ( aes ( y  =  for_eff ,  
                              x =  mov_feroc_stand),  
                          data =  ff)  +  
      facet_wrap ( ~  treat,  scales =   "free_x" )  +  
      geom_point ()  +  
      labs ( x =   "Arousal (coeficient variance of deviations from the feeders/number of feeder changes)" ,  y =    "Foraging efficiency" )  +  
      geom_smooth ( method =   "lm" )  #+  
      # theme_bw();  
     arousalplot 
    
    
    # Explorative behaviour --------------------------------------  
    
    # test  
   model.explr  <-   lmer (for_eff  ~  treat  *   stan_nflo  +  ( 1   |  ID..),  data =  ff,  REML =   FALSE ) 
    summary (model.explr) 
    
    # significance of the random factor  
   model.explr_fix  <-   lm (for_eff  ~  treat  *   stan_nflo,  data =  ff) 
    anova (model.explr, model.explr_fix) 
    
    # plot  
   explot  <-   ggplot ( aes ( y  =  for_eff ,  x =  stan_nflo),  data =  ff)  +  
      facet_wrap ( ~  treat,  scales =   "free_x" )  +   
      geom_point ()  +  
      labs ( x =   "Explorative behavior (# visited feeders/total foraging duration)" ,  y =    "Foraging efficiency" )  +  
      geom_smooth ( method =   "lm" )  #+  
      # theme_bw();  
    
   explot 
    
    # Risk avoidance ------------------------------------------------  
    
    # test  
   model.risk.lat  <-   lmer (for_eff  ~  treat  *   Latency  +  ( 1   |  ID..),  data =  ff,  REML =   FALSE );  summary (model.risk.lat) 
    
    
    # significance of the random factor  
   model.risk.lat_fix  <-   lm (for_eff  ~  treat  *   Latency,  data =  ff) 
    anova (model.risk.lat, model.risk.lat_fix) 
    
    # plot  
   riskplot  <-   ggplot ( aes ( y  =  for_eff ,  x =  Latency),  data =  ff)  +  
      facet_wrap ( ~  treat,  scales =   "free_x" )  +   
      geom_point ()  +  
      labs ( x =   "Risk avoidance (latency)" ,  y =    "Foraging efficiency" )  +  
      geom_smooth ( method =   "lm" )  #+  
      # theme_bw();   
   riskplot 
    
    
    # All behav plots  
    # ggarrange(explot, riskplot, arousalplot,   
    #           labels = c("A", "B", "C"),  
    #           ncol = 1, nrow = 3)  
    # ggsave(filename = "behavplots.tiff", plot = last_plot(),dpi = 300)  
    
    # Repitabilly -------------------------------------------------------------  
    
    # only control group considered  
    library (rptR) 
    
    rpt (mov_feroc_stand  ~  ( 1   |  ID..),  data =  ff[ff $ ConfSimpl  ==   "Ctr" ,],  grname =   "ID.." ,  nboot =   100 ,  npermut =   10 ) 
    
    # rpt(mov_feroc_stand ~ treat + (1 | ID..), data = ff, grname = "ID..", nboot = 100, npermut = 10)  
    
    rpt (stan_nflo  ~  ( 1   |  ID..),  data =  ff[ff $ ConfSimpl  ==   "Ctr" ,],  grname =   "ID.." ,  nboot =   100 ,  npermut =   10 ) 
    
    # rpt(stan_nflo ~ treat + (1 | ID..), data = ff, grname = "ID..", nboot = 100, npermut = 10)  
    
    rpt (Latency  ~  ( 1   |  ID..),  data =  ff[ff $ ConfSimpl  ==   "Ctr" ,],  grname =   "ID.." ,  nboot =   100 ,  npermut =   10 ) 
    
    # rpt(Latency ~ treat + (1 | ID..), data = ff, grname = "ID..", nboot = 100, npermut = 10)  
    
    
    
    # Testing significance of the model estimates - randomization ----------------------  
    
    # basic model  
    
    # Each paramter needs to be processed separately  
    
    # Exploratory behav data  
   df_basic_sel  <-  ff  %>%   select (for_eff, treat, ID.., stan_nflo)  %>%   
      rename ( param =  stan_nflo) 
    
    # Risk-avoidance behav data  
   df_basic_sel  <-  ff  %>%   select (for_eff, treat, ID.., Latency)  %>%   
      rename ( param =  Latency) 
    
    # Arousal behav data  
   df_basic_sel  <-  ff  %>%   select (for_eff, treat, ID.., mov_feroc_stand)  %>%   rename ( param =  mov_feroc_stand) 
    
    ##### START: Common part  
    
    # Basic model for a given parameter  
   basic_model  <-   lmer (for_eff  ~  treat  *   param  +  ( 1   |  ID..),  data =  df_basic_sel,  REML =   FALSE ) 
   basicmodel_sum  <-   summary (basic_model) 
    
    #real coeficients  
   real_treatment  <-  basicmodel_sum $ coefficients[ 2 ] 
   real_parameter  <-  basicmodel_sum $ coefficients[ 3 ] 
   real_interaction  <-  basicmodel_sum $ coefficients[ 4 ] 
    
    
    # Randomization  
    set.seed ( 1313 ) 
    
   N  <-   1000  
    
    # rand_treatment <- numeric()  
    # rand_parameter <- numeric()  
    # rand_interaction <- numeric()  
    #   
    # for (i in 1:N) {  
    #     
    #   df_temp <- df_basic_sel %>% sample_n(size = nrow(df_basic_sel), replace = TRUE)  
    #     
    #   mod_temp <- lmer(for_eff ~ treat *  param + (1 | ID..), data = df_temp, REML = FALSE)  
    #   mod_sum <- summary(mod_temp)  
    #     
    #   rand_treatment[i] <- mod_sum$coefficients[2]  
    #   rand_parameter[i] <- mod_sum$coefficients[3]  
    #   rand_interaction[i] <- mod_sum$coefficients[4]  
    #     
    # }  
    #   
    #   
    # treatment <- rand_treatment  
    # parameter <- rand_parameter  
    # interaction <- rand_interaction  
    #   
    # key <- c(rep("treatment", N),  
    #          rep("parameter", N),  
    #          rep("interaction", N))  
    #   
    # val <- c(treatment, parameter, interaction)  
    #   
    # df <- data.frame(key, val)  
    #   
    # saveRDS(df, "randomization_results_1.RDS")  
    
   df  <-   readRDS ( "./output/randomization_results_1.RDS" ) 
    
    # PLOTs  
    
   rand_parameter  <-  df $ val[df $ key  ==   "parameter" ] 
   rand_treatment  <-  df $ val[df $ key  ==   "treatment" ] 
   rand_interaction  <-  df $ val[df $ key  ==   "interaction" ] 
    # parameter  
    
   pval_param  <-  ( sum (rand_parameter <  0 )) / N 
    
   plot_param  <-   ggplot ( data =  df[df $ key  ==   "parameter" ,])  +   geom_density ( aes ( x =  val),  fill =   "lightgrey" )  +  
      geom_vline ( aes ( xintercept =   0 ),  linetype =   "dashed" )  +   
      geom_vline ( aes ( xintercept =  real_parameter),  col =   "darkblue" ,  size =   1.2 )  +  
      # theme_classic()+  
      scale_x_continuous ( expand =   c ( 0 , 0 ),  name =   paste0 ( "Estimate, P = " , pval_param,  sep =   "" ))  +  
      scale_y_continuous ( expand =   c ( 0 , 0 ),  name =   "Density" ) 
    
    # treatment   
    
   pval_treat  <-  ( sum (rand_treatment >  0 )) / N 
    
   plot_treat  <-   ggplot ( data =  df[df $ key  ==   "treatment" ,],  
                         aes ( x =  val))  +   
      geom_density ( fill =   "lightgrey" )  +   
      geom_vline ( aes ( xintercept =   0 ),  linetype =   "dashed" )  +   
      geom_vline ( aes ( xintercept =  real_treatment),  col =   "darkblue" ,  size =   1.2 )  +  
      # theme_classic()+  
      scale_x_continuous ( expand =   c ( 0 , 0 ),  
                         name =   paste0 ( "Estimate, P = " , pval_treat,  sep =   "" ))  +  
      scale_y_continuous ( expand =   c ( 0 , 0 ),  name =   "Density" )  
    
    # interaction  
    
   pval_inter  <-  ( sum (rand_interaction <  0 )) / N 
    
   plot_inter  <-   ggplot ( data =  df[df $ key  ==   "interaction" ,],  
                         aes ( x =  val))  +   
      geom_density ( fill =   "lightgrey" )  +   
      geom_vline ( aes ( xintercept =   0 ),  linetype =   "dashed" )  +   
      geom_vline ( aes ( xintercept =  real_interaction),  col =   "darkblue" ,  size =   1.2 )  +  
      # theme_classic()+  
      scale_x_continuous ( expand =   c ( 0 , 0 ), limits =   c ( -  0.05 , 0.3 ),  name =   paste0 ( "Estimate, P = " , pval_inter,  sep =   "" ))  +  
      scale_y_continuous ( expand =   c ( 0 , 0 ),  name =   "Density" ) 
    
    
    ggarrange (plot_param, plot_treat, plot_inter,  nrow =   3 ,  labels =   "AUTO" ) 
    
    # ggsave(filename = "C:/Users/KWJ/Dropbox/FF_fear and foraging/explo_random.jpg",   
    #        plot = last_plot(),dpi = 300 )  
    
    # ggsave(filename = "C:/Users/KWJ/Dropbox/FF_fear and foraging/risk_random.jpg",   
    #        plot = last_plot(),dpi = 300 )  
    
    # ggsave(filename = "C:/Users/KWJ/Dropbox/FF_fear and foraging/arousal.jpg",   
    #        plot = last_plot(),dpi = 300 )  
    
    
    
    # behaviours comparison - ctr vs exp -------------------------------------------------  
    
    # data selection  
   df_behav  <-  ff  %>%   
      select (treat, ID.., for_eff, stan_nflo, Latency, mov_feroc_stand)   %>%   
      gather ( key =   "param" ,  value =   "val" ,  -  c (treat, ID..)) 
    
    # test for the significance of the estimates in the model  
    # prm <- unique(df_behav$param)  
    # N <- 1000  
    # EST <- list()  
    #   
    # set.seed(1212)  
    #   
    # for(i in 1:length(prm)) {  
    #     
    #   df_beh <- df_behav %>% filter(param == prm[i])  
    #     
    #   #split data into ctr and exp set to sample separately for the two groups  
    #   df_beh_ctr <- df_beh %>% filter(treat == "Ctr")  
    #   df_beh_exp <- df_beh %>% filter(treat == "Exp")  
    #     
    #   est <- numeric()  
    #     
    #   for (j in 1:N) {  
    #       
    #     df_ctr <- sample_n(df_beh_ctr, nrow(df_beh_ctr), replace = TRUE)  
    #     df_exp <- sample_n(df_beh_exp, nrow(df_beh_exp), replace = TRUE)  
    #     df_behv <- rbind(df_ctr, df_exp)  
    #       
    #     beh_mod <- lmer(data = df_behv, val ~ treat + (1|ID..))  
    #     beh_mod_sum <- summary(beh_mod)  
    #       
    #     est[j] <- beh_mod_sum$coefficients[2]  
    #       
    #   }  
    #     
    #   EST[[i]] <- est  
    # }  
    #   
    #   
    # behav_random <- data.frame(param_rand = rep(prm, each = N))   
    # behav_random$estimate <- c(EST[[1]], EST[[2]], EST[[3]],  EST[[4]])  
    
    # saveRDS(behav_random, "behavior_random_output.RDS")  
    
    # behav_random <- readRDS("./output/behavior_random_output.RDS")  
    
    
    # P value  
   behav_random  %>%   group_by (param_rand)  %>%   
      mutate ( P=   if_else (param_rand  ==   "stan_nflo" , estimate <  0 , 
                        if_else (param_rand  ==   "Latency" ,estimate <  0 , 
                               estimate >  0 )))  %>%   
      summarise ( sum (P) / N) 
    
    
    
    
   df_behav_ind  <-  df_behav  %>%   
      group_by (param, treat, ID..)  %>%   
      summarise ( val =   mean (val))  %>%   
      ungroup ()  %>%   
      mutate ( param =   if_else (param  ==   "mov_feroc_stand" ,  "Arousal" , 
                             if_else (param  ==   "for_eff" ,  "Foraging efficiency" , 
                                     if_else (param  ==   "stan_nflo" ,  "Exporation" ,  "Risk-avoidance" ))))  %>%   
      mutate ( BirdID =   as.factor (ID..) ) 
    
    
    
    
   df_behav  <-  df_behav  %>%   
      mutate ( param =   if_else (param  ==   "mov_feroc_stand" ,  "Arousal" ,  
                             if_else (param  ==   "for_eff" ,  "Foraging efficiency" , 
                                     if_else (param  ==   "stan_nflo" ,  "Exporation" ,  "Risk-avoidance" ))))  %>%   
      mutate ( BirdID =   as.factor (ID..) ) 
    
    
   df_behav $ param_f   <-    factor (df_behav $ param,  
                                 levels=  c ( 'Foraging efficency' , 
                                          'Exploration' , 
                                          'Risk-avoidance' , 
                                          'Arousal' )) 
    
   df_behav_ind $ param_f   <-    factor (df_behav_ind $ param,  
                                     levels=  c ( 'Foraging efficency' , 
                                              'Exploration' , 
                                              'Risk-avoidance' , 
                                              'Arousal' )) 
    
    ggplot ( data =  df_behav,  aes ( x =  treat,  y =  val))  +   
      geom_violin ()  +  
      geom_point ( data =  df_behav_ind,  aes ( x =  treat,  y =  val,  col =  BirdID))  +  
      geom_line ( data =  df_behav_ind,  aes ( x =  treat,  y =  val,  group =  BirdID))   +  
      facet_wrap ( ~ param,  scales =   "free" ,  nrow =   3 )  #+  
      # theme_bw()  
    
    
    ```  
    
    # Variable description (*only those in bold were used in the statistical analysis*):  
    
    -  abs_nflo: absolute number of feeders used (e.g. feeder: A, B, A, B; abs_nflo = 2) 
    -  nflo_chang: number of feeders changes (e.g. feeder: A, B, A, B; nflo_chang = 3) 
    -  nouts: number of "OUTs" foraging breaks (i.e. bill NOT inserted in  the feeder) 
    -  nins: number of "INs" - foraging intervals (i.e. bill inserted in  the feeder) 
    -  mean_durins: mean duration of "INs"    
    -  tot_durins: total duration of the "INs" (i.e. sum of all ins)  
    -  mean_durouts: mean duration of "OUTs"    
    -  tot_durouts: total duration of the "OUTs" (i.e. sum of all ins)   
    -  tot_durfor: total duration of foraging visit (i.e .time between the very first insert and the end of the visit) 
   mov_totdist: total distance covered during the foraging visit 
    -  mov_spead: total distance covered during the foraging visit divided by the total duration of the visit 
    -  mov_feroc: coeficient of variance for the birds position in the 2D space 
    -  ID..: birds ID 
    -  stan_nflochang: time-standardized nflo_change 
    -  stan_nouts: time-standardized nouts 
    -  stan_nins: time-standardized nins 
    -  stand_totdist: time-standardized totdist 
    -  **stan_nflo: time-standardized abs_nflo (i.e. abs_nflo/tot_durfor) *(PROXY FOR EXPLORATIONS)* **     
    -  **for_eff: foraging efficency, i.e. tot_durins / totdurfor** 
    -  **Latency: latency to approach the feeder (i.e. time between birds appearance, like the first hovering in front of the feeder and onset of the visit) *(PROXY FOR RISK AVOIDANCE)* **   
    -  **mov_feroc_stand: time-standardized mov_feroc *(PROXY FOR AROUSAL)* ** 
    
    # Exploring data  
    
    ```{r exploring data, eval = TRUE, warning = FALSE}  
    
    # target variables  
   vars  <-   c ( "stan_nflo" ,  "for_eff" ,  "Latency" ,  "mov_feroc_stand" ) 
    
    # look at data distribution  
   long_foragin_data  <-   do.call (rbind,  lapply (vars,  function (x)  data.frame ( var =  x,  value =  foraging_data[,  names (foraging_data)  ==  x,  drop =   TRUE ]))) 
    
    ggplot (long_foragin_data,  aes (var, value))  +   
      geom_violin ( fill =  cols[ 9 ])  +  
      coord_flip ()  +   
      ggtitle ( "Raw parameters" )  +  
      labs ( x =   "Parameter" ,  y =   "Raw value" ) 
    
    # log transformed  
    ggplot (long_foragin_data,  aes (var,  log (value  +   1 )))  +   
      geom_violin ( fill =  cols[ 9 ])  +  
      coord_flip ()  +   
      ggtitle ( "Log-transformed parameters" )  +  
        labs ( x =   "Parameter" ,  y =   "Log value" ) 
    
    # log transform variables  
   foraging_data $ arousal  <-   log (foraging_data $ mov_feroc_stand  +   1 ) 
   foraging_data $ exploration  <-   log (foraging_data $ stan_nflo  +   1 ) 
   foraging_data $ risk_avoidance  <-   log (foraging_data $ Latency  +   1 ) 
   foraging_data $ foraging_efficiency  <-   log (foraging_data $ for_eff  +   1 ) 
    
   foraging_data $ context  <-   ifelse (foraging_data $ treat  ==   "Ctr" ,  "Low risk" ,  "High risk" ) 
    
   foraging_data $ context  <-   factor (foraging_data $ context,  levels =   c ( "Low risk" ,  "High risk" )) 
    
    # new target variables  
   vars  <-   c ( "exploration" ,  "risk_avoidance" ,  "arousal" ) 
    
    # correlation matrix  
   cm  <-   cor (foraging_data[, vars],  use =   "pairwise.complete.obs" ) 
    
    # visualize collinearity  
    corrplot.mixed (cm,  upper =   "ellipse" ,  lower =   "number" ,  tl.pos =   "lt" ,  upper.col =  col.crrplt,  lower.col =  col.crrplt,  tl.col =   "black" ,  tl.cex =   2 ) 
    
    ```  
    
    <div   class  =  "alert alert-info"  >  
    
    *  Long right tails in distributions (better to log!) 
      
    *  Personality parameters were log-tranformed and renamed: 
        -  log(stan_nflo) -> **exploration**     
        -  log(for_eff) -> **foraging_efficiency**  
        -  Log(Latency) -> **risk_avoidance** 
        -  Log(mov_feroc_stand) -> **arousal** 
          
    *  Little collinearity between predictors 
    
    </div>  
    
    
    # Repeatability  
    
    ```{r Repeatability, warning=FALSE, eval = FALSE}  
    
    pboptions ( type =   "none" ) 
    # rep movement  
    
   rpt_arousal  <-   rpt (arousal  ~  ( 1   |  indiv),  data =  foraging_data[foraging_data $ context  ==   "Low risk" ,],  grname =   "indiv" ,  nboot =   100 ,  npermut =   100 ,  parallel =   TRUE ) 
    
   rpt_exploration  <-   rpt (exploration  ~  ( 1   |  indiv),  data =  foraging_data[foraging_data $ context  ==   "Low risk" ,],  grname =   "indiv" ,  nboot =   100 ,  npermut =   100 ,  parallel =   TRUE ) 
    
   rpt_risk  <-   rpt (risk_avoidance  ~  ( 1   |  indiv),  data =  foraging_data[foraging_data $ context  ==   "Low risk" ,],  grname =   "indiv" ,  nboot =   100 ,  npermut =   100 ,  parallel =   TRUE ) 
    
   rpt_foraging_efficiency  <-   rpt (foraging_efficiency  ~  ( 1   |  indiv),  data =  foraging_data[foraging_data $ context  ==   "Low risk" ,],  grname =   "indiv" ,  nboot =   100 ,  npermut =   100 ,  parallel =   TRUE ) 
    
    
    saveRDS ( list ( arousal =  rpt_arousal,  exploration =  rpt_exploration,  risk_avoidance =  rpt_risk,  foraging_efficiency =  rpt_foraging_efficiency),  "./output/Repeatability results.RDS" ) 
    
    ```  
    
    ```{r plot repeatabilty, warning=FALSE}  
    
   rept  <-   readRDS ( "./output/Repeatability results.RDS" ) 
    
   reps  <-   lapply ( 1  :  length (rept),  function (x){ 
      
     X  <-  rept[[x]] 
      data.frame ( param =   names (rept)[x],  R =  X $ R[ 1 ,],  low.CI =  X $ CI_emp[ 1 ,  1 ],  hi.CI =  X $ CI_emp[ 1 ,  2 ]) 
   }) 
    
   reps.df  <-   do.call (rbind,reps) 
    
    ggplot (reps.df,  aes ( x =  param,  y =  R))  +  
      geom_hline ( yintercept =   0 ,  lty =   2 )  +  
      geom_point ( col =  cols[ 7 ],  size =   5 )  +  
      geom_errorbar ( aes ( ymin =  low.CI,  ymax =  hi.CI),  width= . 0 ,  col =  cols[ 7 ],  size =   2 )  +  
      coord_flip ()   +   labs ( y =   "Repeatability" ,  x =  "Parameters" ) 
    
    ```  
    
    <div   class  =  "alert alert-info"  >  
    
    *  Medium to low repeatability  
    
    *  Non-significant repeatability for arousal 
        
    </div>  
    
    
    # MCMCglmm mixed-effect models  
    
   Bayesian MCMC generalized linear models to predict foraging efficiency with personaltiy-related parameters and their interaction with context (low or high risk) as predictors and individual as a random effect. 
    
   We used two modeling approaches. In the first one ( [  "single predictor approach"  ](#single-predictors) ) indenpendent model selection procedures were run for each personality-parameter. In the second approach a  [  single global model  ](#single-model)  containing all 3 interactions was compared against submodels containing 1 and 2 interaction. In both cases all model selection procedures included the "classical" hypothesis model that ignores within individual variation (so only risk level as predictor). 
    
    <!-- <div class="alert alert-success" role="alert"> -->  
    
   --- 
    
    ## Single predictor models {#single-predictors}  
    
   Three models were compared for each parameter:  
    
    
      1.  only context as predictor (i.e. **"classical" hypothesis**): 
    
   $$foraging\ efficiency \sim context + (1 | indiv)$$ 
    
      2.  context, personality parameters and their interaction as predictors (**alternative hypothesis accounting for individual differences**):  
    
   $$foraging\ efficiency \sim context * personality\ parameter + (1 | indiv)$$ 
    
      3.  Null model with no predictor: 
    
   $$foraging\ efficiency \sim 1 + (1 | indiv)$$ 
    
   --- 
    
    <!-- </div> -->  
    
   A loop is used to run these 3 models for each selected acoustic parameters. Each model is replicated 3 times with starting values sampled from a Z-distribution ("start" argument in MCMCglmm()) and mean-centered so intercept is found at the mean of the predictor variable. Parameters are scaled (i.e. z-transformed)  to obtained standardized effect sizes (within the loop). Diagnostic plots for MCMC model performance are shown at the end of this report: 
    
    ```{r mcmcglmm models, eval = FALSE}  
    
   itrns  <-   100000  
   burnin  <-   10000  
    # null model  
   mcmc_output  <-   pblapply ( c ( "arousal" ,  "exploration" ,  "risk_avoidance" ),  cl =   detectCores ()  -  1 ,  function (x){ 
    
     foraging_subdata  <-  foraging_data[,  c (x,  "indiv" ,  "foraging_efficiency" ,  "context" )] 
      
     foraging_subdata  <-  foraging_subdata[ complete.cases (foraging_subdata[, x]), ] 
      
      # mean centering  
     foraging_subdata[, x]  <-  foraging_subdata[, x]  -   mean (foraging_subdata[, x,  drop =   TRUE ],  na.rm =   TRUE ) 
      
      
     md_null  <-   replicate ( 3 ,  MCMCglmm ( formula ( "foraging_efficiency ~ 1" ),  random =   ~  indiv,  data =  foraging_subdata,  verbose =   FALSE ,  nitt =  itrns,   start =   list ( QUASI =   FALSE ),  burnin =  burnin),  simplify =   FALSE ) 
      
     md_only_context  <-   replicate ( 3 ,  MCMCglmm ( formula ( "foraging_efficiency ~ context" ),  random =   ~  indiv,  data =  foraging_subdata,  verbose =   FALSE ,  nitt =  itrns,   start =   list ( QUASI =   FALSE ),  burnin =  burnin),  simplify =   FALSE ) 
    
     md_only_parameter  <-   replicate ( 3 ,  MCMCglmm ( formula ( paste ( "foraging_efficiency ~" , x)),  random =   ~  indiv,  data =  foraging_subdata,  verbose =   FALSE ,  nitt =  itrns,   start =   list ( QUASI =   FALSE ),  burnin =  burnin),  simplify =   FALSE ) 
        
     md_interation  <-   replicate ( 3 ,  MCMCglmm ( formula ( paste ( "foraging_efficiency ~ context *" , x)),  random =   ~  indiv,  data =  foraging_subdata,  verbose =   FALSE ,  nitt =  itrns,   start =   list ( QUASI =   FALSE ),  burnin =  burnin),  simplify =   FALSE ) 
      
      # put together the first models  
     msDIC  <-   model.sel (md_null[[ 1 ]], md_only_context[[ 1 ]], md_only_parameter[[ 1 ]], md_interation[[ 1 ]],  rank =   "DIC" ) 
      
       # rename delta and weight  
      names (msDIC)[ names (msDIC)  %in%   c ( "delta" ,  "weight" )]  <-   paste0 ( "DIC." ,  c ( "delta" ,  "weight" )) 
      
      # put together the first models  
     msAIC  <-   model.sel (md_null[[ 1 ]], md_only_context[[ 1 ]], md_only_parameter[[ 1 ]], md_interation[[ 1 ]],  rank =   "AIC" ) 
      
       # rename delta and weight  
      names (msAIC)[ names (msAIC)  %in%   c ( "delta" ,  "weight" )]  <-   paste0 ( "AIC." ,  c ( "delta" ,  "weight" )) 
      
     ms  <-   cbind (msDIC, msAIC[,  c ( "AIC" ,  "AIC.delta" ,  "AIC.weight" )]) 
        
      # rename rows so they match predictor names  
      rownames (ms)  <-    gsub ( "[[1]]" ,  "" , rownames (ms),  fixed =   TRUE ) 
      
      # save models in a list  
     res  <-   list ( model.tab =  ms,  md_only_context =  md_only_context,  md_only_parameter =  md_only_parameter,  md_interation =  md_interation,  md_null =  md_null) 
     }) 
    
    names (mcmc_output)  <-   c ( "arousal" ,  "exploration" ,  "risk_avoidance" ) 
    
    saveRDS (mcmc_output,  "model_selection_predict_foraging_efficiency.RDS" ) 
    
    
    ```  
    
    ## Model selection results  
    
    
    -  ordered by delta DIC (but AIC produces equivalent results) 
    -  best model for each parameters is highlighted in green 
    
    ```{r model selection results, warning=FALSE}  
    
   mcmc_output  <-   readRDS ( "./output/model_selection_predict_foraging_efficiency.RDS" ) 
    
    # put all model selection results in a list  
   mod.list  <-   lapply ( 1  :  length (mcmc_output),  function (i)   data.frame ( response =   names (mcmc_output)[i],  predictors =   rownames (mcmc_output[[i]][[ 1 ]]),  as.data.frame (mcmc_output[[i]][[ 1 ]])[,  5  :  12 ],  stringsAsFactors =   FALSE )) 
    
    # make a data frame with all results  
   mod.sel.tab  <-   do.call (rbind, mod.list) 
    
    # rename predictors for table   
   mod.sel.tab $ predictors[ grep ( "interation" ,  mod.sel.tab $ predictors)]  <-   "Context interaction"  
   mod.sel.tab $ predictors[ grep ( "null" ,  mod.sel.tab $ predictors)]  <-   "Null"  
   mod.sel.tab $ predictors[ grep ( "only_context" ,  mod.sel.tab $ predictors)]  <-   "Context"  
   mod.sel.tab $ predictors[ grep ( "only_parameter" ,  mod.sel.tab $ predictors)]  <-   "Parameter"  
    
    
   mod.sel.tab $ DIC.delta  <-   round (mod.sel.tab $ DIC.delta,  2 ) 
   mod.sel.tab $ DIC.weight  <-   round (mod.sel.tab $ DIC.weight,  2 ) 
   mod.sel.tab $ AIC.delta  <-   round (mod.sel.tab $ AIC.delta,  2 ) 
   mod.sel.tab $ AIC.weight  <-   round (mod.sel.tab $ AIC.weight,  2 ) 
    
    options ( knitr.kable.NA =   '' ) 
    
   df1  <-  knitr ::  kable (mod.sel.tab[,  c ( "response" ,  "predictors" ,  "df" ,  "DIC" ,  "DIC.delta" ,  "DIC.weight" ,  "AIC" ,  "AIC.delta" ,  "AIC.weight" )],  row.names =   FALSE ,  escape =   FALSE ,  format =   "html" ) 
    
   df1  <-   row_spec (df1,  which (mod.sel.tab $ DIC.delta ==   0 ),  background =   adjustcolor (cols[ 9 ],  alpha.f =   0.3 )) 
    
    kable_styling (df1,  bootstrap_options =   c ( "striped" ,  "hover" ,  "condensed" ,  "responsive" ),  full_width =   FALSE ,  font_size =   15 ) 
    
    ```  
    
    <div   class  =  "alert alert-info"  >  
    
    *  All best models contained an interaction with a personality parameter 
    
    *  All models with interaction provided a better fit than the context (low vs high risk) models 
        
    </div>  
    
    
   Plot effect sizes by response variable (only models that improved fit compared to the null models are evaluated): 
    
    ```{r effect sizes, eval = TRUE, warning=FALSE, message=FALSE}  
    
    # select best models based on BIC  
   best_mods  <-   lapply (mcmc_output,  function (X){  
      
      # if best model was at least 2 BIC units higher than null  
      if  (X[[ 1 ]][ "md_null" ,  "DIC.delta" ]  >   2 )  
        return (X[[  rownames (X[[ 1 ]])[ 1 ] ]][[ 1 ]])  else  
          return ( NA )  # else if models were as good as null model return NA  
     }) 
    
    # rename  
    names (best_mods)  <-   names (mcmc_output) 
    
    # remove the NA ones (the ones in which the null model was the best)  
   best_mods  <-  best_mods[ sapply (best_mods, class)  ==   "MCMCglmm" ] 
      
    # extract fixed effect size  
   out  <-   lapply ( 1  :  length (best_mods),  function (x){ 
      
      # fixed effects  
     fe  <-   summary (best_mods[[x]]) $ solutions 
    
      # Confidence intervals  
     ci  <-   HPDinterval (best_mods[[x]] $ Sol) 
      
      # sample sizes    
     obs  <-  foraging_data[ complete.cases (foraging_data[ ,  names (best_mods)[x]]), ] 
      
      # put results together in a data frame  
     res  <-   data.frame ( 
        stringsAsFactors =   FALSE ,  
        # response variable name  
        response =   "foraging effiency" ,  
        # personality parameter  
        parameter =   names (best_mods)[x], 
        # predictor name  
        predictor =   rownames (ci)[ 2  :  nrow (ci)],  
        effect_size =  fe[ -  1 ,  "post.mean" ],  
        # lower confident interval  
        CI_2.5 =  ci[ 2  :  nrow (ci),  1 ],  
        # upper confident interval  
        CI_97.5 =  ci[ 2  :  nrow (ci),  2 ],  
        # p value  
        pMCMC  =  fe[ -  1 ,  "pMCMC" ],  
        #intercept  
        intercept =  fe[ 1 ,  "post.mean" ], 
        # number of individuals  
        n.indv =   length ( unique (obs $ indiv)),  
        # number of observations  
        n.obs =   nrow (obs),  
        # mean response  
        mean =   mean (obs[,  names (best_mods)[x],  drop =   TRUE ],  na.rm =   TRUE ),  
        # standard deviation of response  
        sd =   sd (obs[,  names (best_mods)[x],  drop =   TRUE ],  na.rm =   TRUE ) 
       ) 
      
     return (res) 
   }) 
    
    # put effect sizes in a single data frame   
   effect_size_single_preds  <-   do.call (rbind, out) 
    rownames (effect_size_single_preds)  <-   1  :  nrow (effect_size_single_preds) 
    
    
   md  <-  effect_size_single_preds[,  !  grepl ( "mean|sd" ,  names (effect_size_single_preds))] 
    
   md $ CI_2 .5   <-   round (md $ CI_2 .5 ,  4 ) 
   md $ CI_97 .5   <-   round (md $ CI_97 .5 ,  4 ) 
    
    # get the ones that do not overlap with 0  
   mltp  <-  md $ CI_2 .5   *  md $ CI_97 .5  
    
   md $ CI_2 .5   <-   ifelse (mltp  >   0 ,  cell_spec (md $ CI_2 .5 ,  "html" ,  color =  "white" ,  background =  cols[ 7 ],  bold =  T,   font_size =   12 ),   cell_spec (md $ CI_2 .5 ,  "html" )) 
    
   md $ CI_97 .5   <-   ifelse (mltp  >   0 ,  cell_spec (md $ CI_97 .5 ,  "html" ,  color =  "white" ,  background =  cols[ 7 ],  bold =  T,   font_size =   12 ),   cell_spec (md $ CI_97 .5 ,  "html" )) 
    
   df1  <-  knitr ::  kable (md,  row.names =   FALSE ,  escape =   FALSE ,  format =   "html" ,  digits =   c ( 4 )) 
    
   df1  <-   row_spec (df1,  which (mltp  >   0 ),  background =   adjustcolor (cols[ 9 ],  alpha.f =   0.3 )) 
      
    kable_styling (df1,  bootstrap_options =   c ( "striped" ,  "hover" ,  "condensed" ,  "responsive" ),  full_width =   FALSE ,  font_size =   12 ) 
    
    ```  
    
    ### Effect sizes (on foraging efficiency) for interaction terms  
    ```{r effect size graph, warning=FALSE, message=FALSE}  
    
    # get high prob density intervals  
   hpd.mcmc.l  <-   lapply ( 1  :  length (best_mods),  function (x){ 
     
     hpd.mcmcs  <-   HPD_mcmc (best_mods[[x]] $ Sol) 
    
      return (hpd.mcmcs) 
     }) 
    
   hpd.mcmcs  <-   do.call (rbind, hpd.mcmc.l) 
    
    # remove ohter parameters  
   hpd.mcmcs  <-  hpd.mcmcs[ grep ( "risk$" , hpd.mcmcs $ predictor,  invert =   TRUE ), ] 
    
    # context model  
   contextHDP  <-   HPD_mcmc (mcmc_output $ arousal $ md_only_context[[ 1 ]] $ Sol) 
    
   hpd.mcmcs  <-   rbind (hpd.mcmcs, contextHDP) 
    
   hpd.mcmcs $ predictor  <-   gsub ( "context" ,  "" ,  hpd.mcmcs $ predictor) 
    
    
   single_pred_dat  <-  hpd.mcmcs[ grep ( "risk:|risk$" , hpd.mcmcs $ predictor), ] 
    
   gg_single_pred  <-   ggplot ( data =  single_pred_dat)  +   
      geom_vline ( xintercept =   0 ,  lty =   2 )  +  
      geom_density_ridges ( aes ( y =  predictor,  x =  effect_size),  fill =  cols[ 8 ],  alpha =   0.6 )  +   
      scale_y_discrete ( expand =   c ( 0.01 ,  0 ))  +  
      scale_x_continuous ( expand =   c ( 0.01 ,  0 ))  +  
      labs ( x =   "Effect size" ,  y =   "Interaction" ) 
    
   gg_single_pred 
    ```  
    
    ### Foraging efficiency and context  
    
    ```{r}  
   agg_dat  <-   aggregate (foraging_efficiency  ~  context, foraging_data, mean) 
    #   
    # ggplot(foraging_data, aes(x = context, y = foraging_efficiency)) +   
    #  geom_violin(fill = cols[7]) +  
    #   geom_point(data = agg_dat, size = 4, color = cols[2]) +  
    #   labs(x = "Context", y = "Foraging efficiency")  
    #   
    
   cols  <-   viridis ( 10 ) 
    
   agg_dat $ n  <-   sapply ( 1  :  nrow (agg_dat),  function (x)  length ( unique (foraging_data $ indiv[foraging_data $ context  ==  agg_dat $ context[x]])))  
   agg_dat $ n.labels  <-   paste ( "n =" , agg_dat $ n) 
    # agg_dat$sensory_input <- factor(agg_dat$sensory_input)  
    # raincoud plot:  
   fill_color  <-   adjustcolor ( "#e85307" ,  0.6 ) 
    
    ggplot (foraging_data,  aes ( x =  context,  y =  foraging_efficiency))  +  
      # add half-violin from {ggdist} package  
     ggdist ::  stat_halfeye ( 
        fill =  fill_color, 
        alpha =   0.5 , 
        # custom bandwidth  
        adjust =  . 5 , 
        # adjust height  
        width =  . 6 , 
        .width =   0 , 
        # move geom to the cright  
        justification =   - . 2 , 
        point_colour =   NA  
     )  +  
      geom_boxplot ( fill =  fill_color, 
        width =  . 15 , 
        # remove outliers  
        outlier.shape =   NA   # `outlier.shape = NA` works as well  
     )  +  
      # add justified jitter from the {gghalves} package  
     gghalves ::  geom_half_point ( 
        color =  fill_color, 
        # draw jitter on the left  
        side =   "l" , 
        # control range of jitter  
        range_scale =  . 4 , 
        # add some transparency  
        alpha =  . 5 , 
     )  +     
      ylim ( c ( 0 ,  0.75 ))  +  
      geom_text ( data =  agg_dat,  aes ( y =   rep ( 0.01 ,  2 ),  x =  context,  label =  n.labels),  nudge_x =   0 ,  size =   6 )  +   
       # scale_x_discrete(labels=c("Control" = "Noise control", "Sound vision" = "Sound & vision", "Vision" = "Vision", "Lessen input" = "Lessen input")) +  
      labs ( x =   "Context" ,  y =   "Foraging efficiency" )  
    
    ```  
    
    
    ### Scatter plots with best fit lines   
    
    ```{r scatter plots single predictors, warning = FALSE, message = FALSE, fig.height = 16, fig.width = 14}  
   cols  <-   rep (cols[ 7 ],  10 ) 
    
   out  <-   lapply ( names (best_mods),  function (x){ 
      
     mod  <-  best_mods[[x]] 
       
     pred  <-   predict.MCMCglmm (mod,  interval =   "confidence" ) 
      
     rep_dat  <-   cbind (foraging_data[ !  is.na (foraging_data[, x,  drop =   TRUE ]), ], pred) 
      
      ### both data sets in a single plot  
      # ggplot(rep_dat, aes(x = exploration, y = foraging_efficiency, color = context)) +  
      #   geom_ribbon(aes(ymin = lwr, ymax = upr, fill = context), alpha = .1, show.legend = FALSE, lwd = 0) +  
      #     geom_line(aes(y = fit), size = 1) +  
      #   scale_color_manual(values = cols[c(3, 8)]) +  
      #   geom_point(size = 2) +  
      #   labs(x = "log(exploratory behavior)", y = "Foraging efficiency") +  
      #   theme(legend.position = c(0.8, 0.7), legend.background = element_rect("transparent"))  
      
     gg_hi  <-   ggplot (rep_dat[rep_dat $ context  ==   "High risk" , ],  aes ( x =   get (x),  y =  foraging_efficiency,  color =  context))  +  
        geom_ribbon ( aes ( ymin =  lwr,  ymax =  upr,  fill =  context),  alpha =  . 2 ,  lwd =   0 )  +  
          geom_line ( aes ( y =  fit),  size =   1.5 )  +  
        scale_color_manual ( values =  cols[ 3 ])  +  
        geom_point ( size =   3 )  +  
      labs ( x =   paste0 ( "log(" ,  gsub ( "_" ,  " " , x),  ")" ),  y =   "" )  +   
            theme_classic ( base_size =   20 )  +  
        theme ( legend.position =   "none" ,  axis.text.y =   element_blank (),  axis.ticks.y =   element_blank ()) 
      
     gg_lo  <-   ggplot (rep_dat[rep_dat $ context  !=   "High risk" , ],  aes ( x =   get (x),  y =  foraging_efficiency,  color =  context))  +  
        geom_ribbon ( aes ( ymin =  lwr,  ymax =  upr,  fill =  context),  alpha =  . 2 ,  lwd =   0 )  +  
          geom_line ( aes ( y =  fit),  size =   1.5 )  +  
        scale_color_manual ( values =  cols[ 8 ])  +  
        geom_point ( size =   3 )  +  
          labs ( x =   paste0 ( "log(" ,  gsub ( "_" ,  " " , x),  ")" ),  y =   "Foraging efficiency" )  +  
        theme_classic ( base_size =   20 )  +  
        theme ( legend.position=  "none" ,  axis.title.y =   element_blank ()) 
      
      
     return ( list (gg_lo, gg_hi))  
        
   }) 
    
   plot_list  <-   unlist (out,  recursive =   FALSE ) 
    
    
   pg  <-   plot_grid ( plotlist =  plot_list,  ncol =   2 ,  rel_widths =   c ( 1 ,  1 )) 
    
    # title for left low risk  
   t_lo  <-   ggdraw ()  +   
      draw_label ( 
        "Low risk" , 
        fontface =   'bold' , 
        hjust =   0.5 , 
        size =   20  
       ) 
      
    # title for right high risk  
   t_hi  <-   ggdraw ()  +   
      draw_label ( 
        "High risk" , 
        fontface =   'bold' , 
        hjust =   0.5 , 
        size =   20  
     ) 
    
   ptitles  <-   plot_grid (t_lo, t_hi,  ncol =   2 ,  rel_widths =   c ( 1 ,  0.9 )) 
    
   two_colm_plot  <-   plot_grid ( 
     ptitles, pg, 
      ncol =   1 , 
      # rel_heights values control vertical title margins  
      rel_heights =   c ( 0.1 ,  1 ) 
   ) 
    
   t_ylab  <-   ggdraw ()  +   
      draw_label ( 
        "Foraging efficiency" , 
        fontface =   'bold' , 
        hjust =   0.5 , 
        size =   20 , 
        angle =   90  
     ) 
    
    plot_grid ( 
     t_ylab, two_colm_plot, 
      ncol =   2 , 
      # rel_heights values control vertical title margins  
      rel_widths =   c ( 0.05 ,  1 ) 
     ) 
    
    
    
    #######  
    ```  
    
    <div   class  =  "alert alert-info"  >  
    
    *  As expected, foraging efficiency decreases in high risk contexts 
    
    *  Higher arousal is associated with higher foraging efficiency when facing higher risks 
    
    *  Highly explorative behavior is increases foraging efficiency when facing lower risks but decreases efficiency at higher risks 
    
    *  Risk avoidance tend to lower efficiency but does not differ between risk levels 
    
    </div>  
    
   --- 
    
    ## Single global model {#single-model}  
    
    
   Alternatively we can run a single global model that contains all personality parameters and their interaction with context. 
    
    <!-- <div class="alert alert-success" role="alert"> -->  
   --- 
     
    ### Models  
     
    We tried 3 types of models from all posible models of interactions between 'context' and 'personality' parameters, as well as the context only model and the null model: 
     
      1.  context, personality parameters and their interaction as predictors. This included models with 1, 2 and 3 interaction terms (all constitute **alternative hypotheses accounting for individual differences**):  
    
   $$foraging\ efficiency \sim context * person.param1 + (1 | indiv)$$ 
    
   $$foraging\ efficiency \sim context * person.param1 + 
   context * person.param2 + (1 | indiv)$$ 
    
   $$foraging\ efficiency \sim context * person.param1 + context * person.param2 + context * person.param3 + (1 | indiv)$$ 
    
      2.  only context as predictor (i.e. **"classical" hypothesis**): 
   $$foraging\ efficiency \sim context + (1 | indiv)$$ 
    
      3.  Null model with no predictor: 
   $$foraging\ efficiency \sim 1 + (1 | indiv)$$ 
   --- 
     
     <!-- </div> -->  
    
    
    ```{r several predictors single model, eval = FALSE}  
    
   foraging_subdata  <-  foraging_data[,  c ( "arousal" ,  "exploration" ,  "risk_avoidance" ,  "indiv" ,  "foraging_efficiency" ,  "context" )] 
      
   foraging_subdata  <-  foraging_subdata[ complete.cases (foraging_subdata), ] 
      
   itrns  <-   100000  
    
   md_null  <-   replicate ( 3 ,  MCMCglmm (foraging_efficiency  ~   1 ,  random =   ~  indiv,  data =  foraging_subdata,  verbose =   FALSE ,  nitt =  itrns,   start =   list ( QUASI =   FALSE )),  simplify =   FALSE ) 
      
   md_all_interactions  <-   replicate ( 3 ,  MCMCglmm (foraging_efficiency  ~  context * arousal  +  context * exploration  +  context * risk_avoidance,  random =   ~  indiv,  data =  foraging_subdata,  verbose =   FALSE ,  nitt =  itrns,   start =   list ( QUASI =   FALSE )),  simplify =   FALSE ) 
    
   md_arousal_exploration  <-   replicate ( 3 ,  MCMCglmm (foraging_efficiency  ~  context * arousal  +  context * exploration,  random =   ~  indiv,  data =  foraging_subdata,  verbose =   FALSE ,  nitt =  itrns,   start =   list ( QUASI =   FALSE )),  simplify =   FALSE ) 
      
   md_arousal_risk_avoidance  <-   replicate ( 3 ,  MCMCglmm (foraging_efficiency  ~  context * arousal  +  context * risk_avoidance,  random =   ~  indiv,  data =  foraging_subdata,  verbose =   FALSE ,  nitt =  itrns,   start =   list ( QUASI =   FALSE )),  simplify =   FALSE ) 
    
   md_risk_avoidance_exploration  <-   replicate ( 3 ,  MCMCglmm (foraging_efficiency  ~  context * risk_avoidance  +  context * exploration,  random =   ~  indiv,  data =  foraging_subdata,  verbose =   FALSE ,  nitt =  itrns,   start =   list ( QUASI =   FALSE )),  simplify =   FALSE ) 
    
    # single interaction models  
   md_arousal  <-   replicate ( 3 ,  MCMCglmm (foraging_efficiency  ~  context * arousal,  random =   ~  indiv,  data =  foraging_subdata,  verbose =   FALSE ,  nitt =  itrns,   start =   list ( QUASI =   FALSE )),  simplify =   FALSE ) 
    
   md_risk_avoidance  <-   replicate ( 3 ,  MCMCglmm (foraging_efficiency  ~  context * risk_avoidance,  random =   ~  indiv,  data =  foraging_subdata,  verbose =   FALSE ,  nitt =  itrns,   start =   list ( QUASI =   FALSE )),  simplify =   FALSE ) 
    
   md_exploration  <-   replicate ( 3 ,  MCMCglmm (foraging_efficiency  ~  context * exploration,  random =   ~  indiv,  data =  foraging_subdata,  verbose =   FALSE ,  nitt =  itrns,   start =   list ( QUASI =   FALSE )),  simplify =   FALSE ) 
    
   md_context  <-   replicate ( 3 ,  MCMCglmm (foraging_efficiency  ~  context,  random =   ~  indiv,  data =  foraging_subdata,  verbose =   FALSE ,  nitt =  itrns,   start =   list ( QUASI =   FALSE )),  simplify =   FALSE ) 
    
      # put together the first models  
     msDIC  <-   model.sel (md_null[[ 1 ]], md_all_interactions[[ 1 ]], md_arousal_exploration[[ 1 ]], md_arousal_risk_avoidance[[ 1 ]], md_risk_avoidance_exploration[[ 1 ]],  
                        md_risk_avoidance[[ 1 ]], md_arousal[[ 1 ]], md_exploration[[ 1 ]],  
                        md_context[[ 1 ]],  rank =   "DIC" ) 
      
      # rename delta and weight  
      names (msDIC)[ names (msDIC)  %in%   c ( "delta" ,  "weight" )]  <-   paste0 ( "DIC." ,  c ( "delta" ,  "weight" )) 
      
     msAIC  <-   model.sel (md_null[[ 1 ]], md_all_interactions[[ 1 ]], md_arousal_exploration[[ 1 ]], md_arousal_risk_avoidance[[ 1 ]], md_risk_avoidance_exploration[[ 1 ]],  
                       md_risk_avoidance[[ 1 ]], md_arousal[[ 1 ]], md_exploration[[ 1 ]],  
                        md_context[[ 1 ]],  rank =   "AIC" ) 
      
      # rename delta and weight  
      names (msAIC)[ names (msAIC)  %in%   c ( "delta" ,  "weight" )]  <-   paste0 ( "AIC." ,  c ( "delta" ,  "weight" )) 
    
     ms  <-   cbind (msDIC, msAIC[,  c ( "AIC" ,  "AIC.delta" ,  "AIC.weight" )]) 
        
      # rename rows so they match predictor names  
      rownames (ms)  <-    gsub ( "[[1]]" ,  "" , rownames (ms),  fixed =   TRUE ) 
      
      # save models in a list  
     res  <-   list ( model.tab =  ms,  md_all_interactions =  md_all_interactions,  md_arousal_exploration =  md_arousal_exploration,  md_arousal_risk_avoidance =  md_arousal_risk_avoidance,  md_risk_avoidance_exploration =  md_risk_avoidance_exploration,  md_risk_avoidance =  md_risk_avoidance,  md_arousal =  md_arousal,  md_exploration =  md_exploration,  md_context =  md_context,  md_null =  md_null) 
    
    saveRDS (res,  "model_selection_all_parameters_foraging_efficiency.RDS" ) 
    
    ```  
    
    ### Model selection  
    ```{r model selection results single model}  
    
   mcmc_output  <-   readRDS ( "./output/model_selection_all_parameters_foraging_efficiency.RDS" ) 
    
    # make a data frame with all results  
   mod.sel.tab  <-   data.frame ( response =   "Foraging efficiency" ,  predictors =   rownames (mcmc_output[[ 1 ]]),  as.data.frame (mcmc_output[[ 1 ]]),  stringsAsFactors =   FALSE ) 
    
    # rename predictors for table   
    rownames (mod.sel.tab)  <-   gsub ( "md_" ,  "" ,  rownames (mod.sel.tab)) 
    
   mod.sel.tab $ DIC.delta  <-   round (mod.sel.tab $ DIC.delta,  2 ) 
   mod.sel.tab $ DIC.weight  <-   round (mod.sel.tab $ DIC.weight,  2 ) 
   mod.sel.tab $ AIC.delta  <-   round (mod.sel.tab $ AIC.delta,  2 ) 
   mod.sel.tab $ AIC.weight  <-   round (mod.sel.tab $ AIC.weight,  2 ) 
    
    options ( knitr.kable.NA =   '' ) 
    
   df1  <-  knitr ::  kable (mod.sel.tab[,  c ( "response" ,  "predictors" ,  "df" ,  "DIC" ,  "DIC.delta" ,  "DIC.weight" ,  "AIC" ,  "AIC.delta" ,  "AIC.weight" )],  row.names =   FALSE ,  escape =   FALSE ,  format =   "html" ) 
    
   df1  <-   row_spec (df1,  which (mod.sel.tab $ DIC.delta ==   0 ),  background =   adjustcolor (cols[ 9 ],  alpha.f =   0.3 )) 
    
    kable_styling (df1,  bootstrap_options =   c ( "striped" ,  "hover" ,  "condensed" ,  "responsive" ),  full_width =   FALSE ,  font_size =   11 ) 
    
    ```  
    
    <div   class  =  "alert alert-info"  >  
    
    *  Best model includes all interactions 
    
    </div>  
    
    ### Effect sizes for best model  
    ```{r effect sizes single model, eval = TRUE}  
    
    # select best models based on BIC  
   best_mod  <-  mcmc_output[[ 2 ]] 
    
    # fixed effects  
   fe  <-   summary (best_mod[[ 1 ]]) $ solutions 
    
    # Confidence intervals  
   ci  <-   HPDinterval (best_mod[[ 1 ]] $ Sol) 
    
    # observations used  
   obs  <-  foraging_data[ complete.cases (foraging_data[,  c ( "arousal" ,  "exploration" ,  "risk_avoidance" ,  "indiv" ,  "foraging_efficiency" ,  "context" )]), ] 
      
    # put results together in a data frame  
   effect_size_single_model  <-   data.frame ( 
      stringsAsFactors =   FALSE ,  
      # response variable name  
      response =   "foraging effiency" ,  
      # predictor name  
      predictor =   rownames (ci)[ 2  :  nrow (ci)],  
      effect_size =  fe[ -  1 ,  "post.mean" ],  
      # lower confident interval  
      CI_2.5 =  ci[ 2  :  nrow (ci),  1 ],  
      # upper confident interval  
      CI_97.5 =  ci[ 2  :  nrow (ci),  2 ],  
      # p value  
      pMCMC  =  fe[ -  1 ,  "pMCMC" ],  
      #intercept  
      intercept =  fe[ 1 ,  "post.mean" ], 
      # number of individuals  
      n.indv =   length ( unique (obs $ indiv)),  
      # number of observations  
      n.obs =   nrow (obs) 
   ) 
      
    rownames (effect_size_single_model)  <-   1  :  nrow (effect_size_single_model) 
    
    
   md  <-  effect_size_single_model[,  !  grepl ( "mean|sd" ,  names (effect_size_single_model))] 
    
   md $ CI_2 .5   <-   round (md $ CI_2 .5 ,  4 ) 
   md $ CI_97 .5   <-   round (md $ CI_97 .5 ,  4 ) 
    
    # get the ones that do not overlap with 0  
   mltp  <-  md $ CI_2 .5   *  md $ CI_97 .5  
    
   md $ CI_2 .5   <-   ifelse (mltp  >   0 ,  cell_spec (md $ CI_2 .5 ,  "html" ,  color =  "white" ,  background =  cols[ 7 ],  bold =  T,   font_size =   12 ),   cell_spec (md $ CI_2 .5 ,  "html" )) 
    
   md $ CI_97 .5   <-   ifelse (mltp  >   0 ,  cell_spec (md $ CI_97 .5 ,  "html" ,  color =  "white" ,  background =  cols[ 7 ],  bold =  T,   font_size =   12 ),   cell_spec (md $ CI_97 .5 ,  "html" )) 
    
   df1  <-  knitr ::  kable (md,  row.names =   FALSE ,  escape =   FALSE ,  format =   "html" ,  digits =   c ( 4 )) 
    
   df1  <-   row_spec (df1,  which (mltp  >   0 ),  background =   adjustcolor (cols[ 9 ],  alpha.f =   0.3 )) 
      
    kable_styling (df1,  bootstrap_options =   c ( "striped" ,  "hover" ,  "condensed" ,  "responsive" ),  full_width =   FALSE ,  font_size =   12 ) 
    
    ```  
    
    <div   class  =  "alert alert-info"  >  
    
    *  Similar to single predictor models 
    
    *  Risk avoidance doesn't affect foraging efficiency 
    
    </div>  
    
    ### Effect sizes (on foraging efficiency) for interaction terms  
    ```{r effect size graph single model, warning=FALSE, message=FALSE}  
    #   
    # effect_size_single_model$predictor <- gsub("context", "", effect_size_single_model$predictor)  
    #   
    # ggplot(effect_size_single_model[grep("risk:", effect_size_single_model$predictor), ], aes(x = predictor, y = effect_size)) +   
    #   geom_hline(yintercept = 0, lty = 2) +  
    #   geom_point(col = cols[7], size = 5) +  
    #   geom_errorbar(aes(ymin=CI_2.5, ymax=CI_97.5), width= 0, col = cols[7], size = 2) +  
    #   coord_flip()  
    
   hpd.mcmcs  <-   HPD_mcmc (best_mod[[ 1 ]] $ Sol) 
    
    # remove other context predictors  
   hpd.mcmcs  <-  hpd.mcmcs[hpd.mcmcs $ predictor  !=   "contextHigh risk" , ] 
    
    # add context  
   hpd.mcmcs.context  <-   HPD_mcmc (mcmc_output $ md_context[[ 1 ]] $ Sol) 
    
   hpd.mcmcs  <-   rbind (hpd.mcmcs, hpd.mcmcs.context) 
    
    
   hpd.mcmcs $ predictor  <-   gsub ( "context" ,  "" , hpd.mcmcs $ predictor) 
    
   effect_size_single_model $ predictor  <-   gsub ( "context" ,  "" , effect_size_single_model $ predictor) 
    
   single_mod_dat  <-  hpd.mcmcs[ grep ( "risk:|risk$" , hpd.mcmcs $ predictor), ] 
    
   gg_single_mod  <-   ggplot ( data =  single_mod_dat)  +   
      geom_vline ( xintercept =   0 ,  lty =   2 )  +  
      geom_density_ridges ( aes ( y =  predictor,  x =  effect_size),  fill =  cols[ 8 ],  alpha =   0.6 )  +   
      scale_y_discrete ( expand =   c ( 0.01 ,  0 ))  +  
      scale_x_continuous ( expand =   c ( 0.01 ,  0 ))  +  
      labs ( x =   "Effect size" ,  y =   "Interaction" ) 
    
   gg_single_mod 
    
    ```  
    
    <div   class  =  "alert alert-info"  >  
    
   Similar to single predictor models: 
    
    *  Foraging effiency decreases in high risk contexts 
    
    *  Higher arousal is associated with higher foraging efficiency when facing higher risks 
    
    *  Higher exploration is associated with lower foraging efficiency when facing higher risks 
    
    *  Risk avoidance does not affect significantly 
    
    </div>  
    
   Look at estimates from single predictor models and global model: 
    
    ```{r put both effect size plots together, warning=FALSE, message=FALSE}  
    
   single_pred_dat $ models  <-   "single predictor"  
   single_mod_dat $ models  <-   "single model"  
    
   mods_dat  <-   rbind (single_pred_dat, single_mod_dat) 
    
    ggplot ( data =  mods_dat[mods_dat $ predictor  !=   "High risk" , ])  +   
      geom_vline ( xintercept =   0 ,  lty =   2 )  +  
      geom_density_ridges ( aes ( y =  predictor,  x =  effect_size,  fill =  models),   alpha =   0.6 )  +   
      scale_fill_viridis_d ( begin =   0.4 ,  end =   0.9 )  +   
      scale_y_discrete ( expand =   c ( 0.01 ,  0 ))  +  
      scale_x_continuous ( expand =   c ( 0.01 ,  0 ))  +  
        theme ( legend.position =   c ( 0.36 ,  0.9 ))  +  
      labs ( x =   "Effect size" ,  y =   "Interaction" ) 
    
    ```  
    
    
    ```{r scatter plots single model, eval = FALSE, echo = FALSE, warning = FALSE, message = FALSE, fig.height = 6, fig.width = 12}  
    ##NOT WORKING  
   cols  <-   rep (cols[ 7 ],  10 ) 
    
   mod  <-  best_mod[[ 1 ]] 
    
   dat  <-  foraging_data[ complete.cases (foraging_data[,  c ( "arousal" ,  "exploration" ,  "risk_avoidance" ,  "indiv" ,  "foraging_efficiency" ,  "context" )]), ] 
    
   out  <-   lapply ( names (best_mods),  function (x){ 
      
      if  (x  !=   "exploration" ) 
       dat $ exploration  <-   ifelse (dat $ context  ==   "High risk"  ,  mean (dat $ exploration[dat $ context  ==   "High risk" ]),  mean (dat $ exploration[dat $ context  !=   "High risk" ])) 
    
      if  (x  !=   "arousal" ) 
       dat $ arousal  <-   mean (dat $ arousal) 
    
      if  (x  !=   "risk_avoidance" ) 
       dat $ risk_avoidance  <-   mean (dat $ risk_avoidance) 
        
     pred  <-   predict.MCMCglmm (mod,  newdata =  dat[, c ( "exploration" ,  "arousal" ,  "context" ,  "indiv" ,  "foraging_efficiency" ,  "risk_avoidance" )],  interval =   "confidence" ) 
    
     rep_dat  <-   cbind (dat, pred) 
      
      ### both data sets in a single plot  
      # ggplot(rep_dat, aes(x = exploration, y = foraging_efficiency, color = context)) +  
      #   geom_ribbon(aes(ymin = lwr, ymax = upr, fill = context), alpha = .1, show.legend = FALSE, lwd = 0) +  
      #     geom_line(aes(y = fit), size = 1) +  
      #   scale_color_manual(values = cols[c(3, 8)]) +  
      #   geom_point(size = 2) +  
      #   labs(x = "log(exploratory behavior)", y = "Foraging efficiency") +  
      #   theme(legend.position = c(0.8, 0.7), legend.background = element_rect("transparent"))  
      
     gg_hi  <-   ggplot (rep_dat[rep_dat $ context  ==   "High risk" , ],  aes ( x =   get (x),  y =  foraging_efficiency,  color =  context))  +  
        geom_ribbon ( aes ( ymin =  lwr,  ymax =  upr,  fill =  context),  alpha =  . 1 ,  lwd =   0 )  +  
          geom_line ( aes ( y =  fit),  size =   1.5 )  +  
        scale_color_manual ( values =  cols[ 3 ])  +  
        geom_point ( size =   3 )  +  
        labs ( x =   paste0 ( "log(" , x,  ")" ),  y =   "Foraging efficiency" )  +  
        theme ( legend.position=  "none" ) 
      
     gg_lo  <-   ggplot (rep_dat[rep_dat $ context  !=   "High risk" , ],  aes ( x =   get (x),  y =  foraging_efficiency,  color =  context))  +  
        geom_ribbon ( aes ( ymin =  lwr,  ymax =  upr,  fill =  context),  alpha =  . 1 ,  lwd =   0 )  +  
          geom_line ( aes ( y =  fit),  size =   1.5 )  +  
        scale_color_manual ( values =  cols[ 8 ])  +  
        geom_point ( size =   3 )  +  
        labs ( x =   paste0 ( "log(" ,x,  ")" ),  y =   "Foraging efficiency" )  +   
        theme ( legend.position =   "none" ) 
      
     return ( plot_grid (gg_hi, gg_lo,  ncol =   2 ))  
        
   }) 
    
   out 
    
    ```  
    
    <div   class  =  "alert alert-info"  >  
    
    *  Results are consistent despite of the statistical approach 
    
    </div>  
    
    
    ```{r rest of kasia code use or delete, eval = FALSE, echo = FALSE}  
    # Testing significance of the model estimates - randomization ----------------------  
    
    # basic model  
    
    # Each paramter needs to be processed separately  
    
    # Exploratory behav data  
   df_basic_sel  <-  ff  %>%   select (for_eff, treat, ID.., stan_nflo)  %>%   
      rename ( param =  stan_nflo) 
    
    # Risk-avoidance behav data  
   df_basic_sel  <-  ff  %>%   select (for_eff, treat, ID.., Latency)  %>%   
      rename ( param =  Latency) 
    
    # Arousal behav data  
   df_basic_sel  <-  ff  %>%   select (for_eff, treat, ID.., mov_feroc_stand)  %>%   rename ( param =  mov_feroc_stand) 
    
    ##### START: Common part  
    
    # Basic model for a given parameter  
   basic_model  <-   lmer (for_eff  ~  treat  *  param  +  ( 1   |  ID..),  data =  df_basic_sel,  REML =   FALSE ) 
   basicmodel_sum  <-   summary (basic_model) 
    
    #real coeficients  
   real_treatment  <-  basicmodel_sum $ coefficients[ 2 ] 
   real_parameter  <-  basicmodel_sum $ coefficients[ 3 ] 
   real_interaction  <-  basicmodel_sum $ coefficients[ 4 ] 
    
    
    
    ```  
    
    ## Diagnostic stats and plots on MCMCglmm models  
    
    ### Single parameter models  
    ```{r diagnostic MCMCglmm single parameters, eval = TRUE, echo = TRUE, fig.height= 5}  
    
    # read skipping model selection table  
   mcmc_single_param  <-   readRDS ( "./output/model_selection_predict_foraging_efficiency.RDS" ) 
    
    for (w  in   1  :  length (mcmc_single_param)) 
   { 
      print ( paste ( "Predictor:" ,  names (mcmc_single_param)[w])) 
      
     mods  <-  mcmc_single_param[[w]][ -  1 ] 
      
    for (x  in   1  :  length (mods)){ 
    
    print ( names (mods)[x]) 
    
     X  <-  mods[[x]] 
      plot_repl_mcmc_models (X,  begin =   0.4 ) 
     } 
   }       
    ```  
    
    
    ### Global model  
    ```{r diagnostic MCMCglmm global model, eval = TRUE, echo = TRUE, fig.height= 5}  
    
    # read skipping model selection table  
   mcmc_all_param  <-   readRDS ( "./output/model_selection_all_parameters_foraging_efficiency.RDS" )[ -  1 ] 
    
    for (x  in   1  :  length (mcmc_all_param)){ 
    
    print ( names (mcmc_all_param)[x]) 
    
     X  <-  mcmc_all_param[[x]] 
      plot_repl_mcmc_models (X,  begin =   0.4 ) 
   } 
          
    ```  
    
   --- 
    
    <font   size  =  "5"  > Session information </font>  
    
    ```{r session info, echo=F}  
    
    sessionInfo () 
    
    
    ```  
       
     
   


  
