# Supplementary material for: Foraging, Fear and Behavioral Variation in a Traplining Hummingbird
Source: Animals (Basel). 2023 Jun 15;13(12):1997. doi: 10.3390/ani13121997 (PMC10295738; doi:10.3390/ani13121997)
Supplement: Supplementary file 1 [file animals-13-01997-s001.zip › supplementary_S2.pdf]

SUPPLEMENTARY ONLINE MATERIALS: Foraging, fear and behavioural variation in traplining hummingbirds

Effect of time

Katarzyna Wojczulanis-Jakubas, Marcelo Araya-Salas

To analyse the effect of passing time (i.e. habituation, satiation) on the foraging efficency, we examined available time series of foraging efficiency with two approaches. Firstly, we considered a linear mixed model (*lmer* function in *lmerTEST* package, Kuzetsowa et al 2017) with foraging efficiency as a response variable and the sequence of consecutive visits during the control phases as an explanatory (linear for simplicity) response. Birds indentity was also included in the model as a random factor. The model output indicated on significant and positive effect of the visit on the foraging efficiency (glmm, estimate 0.006 ± SE 0.002, t = 1.47, df = 135.3, P = 0.03; Fig S1).

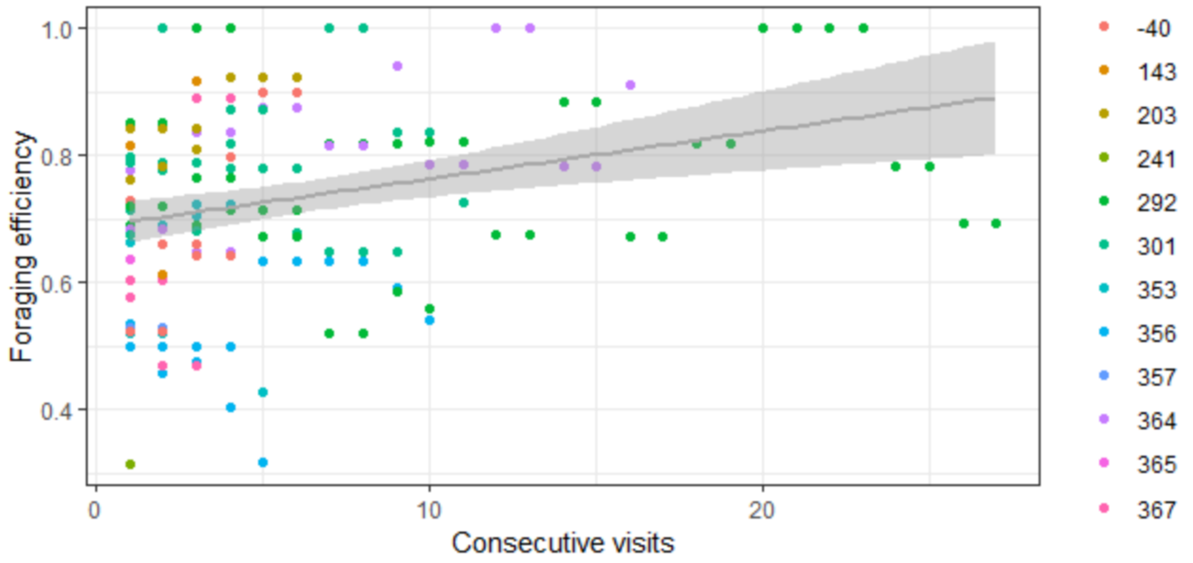

Figure S1 Relationship between foraging efficiency and consecutive visits at feeders area during the control phases of the experiment (all individuals considered).

In the second approach we used data of six time series from four different individuals that were the most frequent visitors at the feeder during control phases (≥ 5 records of consecutive visits during the control phases). We did not find any evidence of the autocorrelation in the examined set, i.e. correlation coeficients for all the lags were statistically insignificant for all the examined time series (Fig. S2).

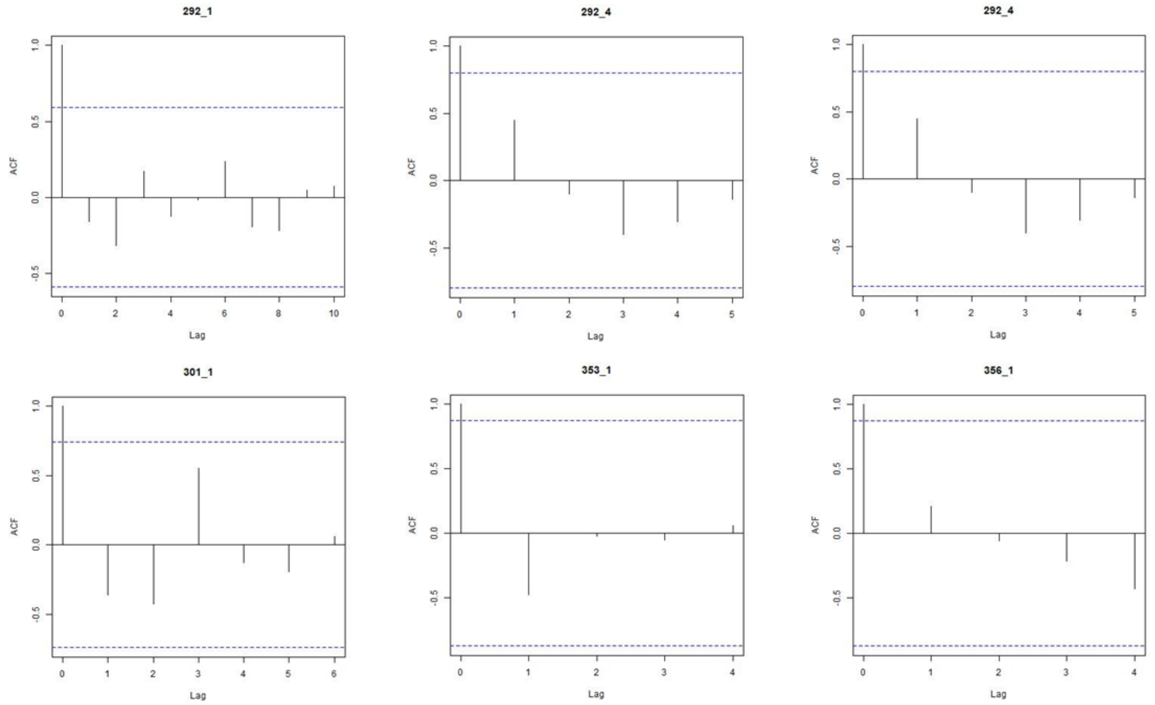

Figure S2 Autocorrelation analysis plots for six time series (four individuals) of foraging efficiency during consecutive visits at feeders area during the control phases of the experiment. The titles denote individual identity and number of control session. Solid vertical lines denote correlation coefficient for particular lag of the time series, and dashed horizontal lines delimit the range of their significance.
